# Understanding Mechanisms of Whole Brain and Regional Grey Matter Atrophy in Children With MOGAD

**DOI:** 10.1002/acn3.70123

**Published:** 2025-07-02

**Authors:** Ermelinda De Meo, Riccardo Nistri, Michael Eyre, Cheryl Hemingway, Ming Lim, Thomas Rossor, Asthik Biswas, Kshitij Mankad, Ata Siddiqui, Sniya Sudhakar, Declan Chard, Frederik Barkhof, Arman Eshaghi, Olga Ciccarelli, Yael Hacohen

**Affiliations:** ^1^ Department of Neuroinflammation, Queen Square MS Centre UCL Queen Square Institute of Neurology London UK; ^2^ NEUROFARBA Department Univeristy of Florence Italy; ^3^ Children's Neurosciences, Evelina London Children's Hospital at Guy's and St Thomas' NHS Foundation Trust London UK; ^4^ Departments of Imaging Physics & Engineering and Early Life Imaging, School of Biomedical Engineering & Imaging Sciences King's College London London UK; ^5^ Department of Women and Children's Health, School of Life Course Sciences (SoLCS) King's College London London UK; ^6^ Department of Neurology Great Ormond Street Hospital for Children NHS Foundation Trust London UK; ^7^ Department of Neuroradiology Great Ormond Street Hospital, Great Ormond Street Hospital Trust London UK; ^8^ Department of Neuroradiology Evelina London Children's Hospital, Guy's and St Thomas' NHS Foundation Trust London UK; ^9^ National Institute for Health and Care Research (NIHR), University College London Hospitals (UCLH), Biomedical Research Centre London UK; ^10^ National Hospital for Neurology and Neurosurgery London UK; ^11^ Department of Medical Physics and Biomedical Engineering and Department of Computer Science, UCL Hawkes Institute University College London London UK; ^12^ Department of Radiology and Nuclear Medicine VU University Medical Centre Amsterdam the Netherlands

**Keywords:** developmental failure, grey matter atrophy, MOGAD

## Abstract

**Objective:**

To investigate the mechanisms driving whole brain and regional grey matter (GM) volume changes along with their clinical correlates in paediatric myelin oligodendrocyte glycoprotein antibody (MOG‐Ab)–associated disease (MOGAD).

**Methods:**

One‐hundred‐nine paediatric MOGAD patients from two UK centres underwent MRI at attack nadir and follow‐up (at least 1) ≥ 6 weeks later. Normative trajectories from 317 typically developing children informed volumetric comparisons. MRI segmentation with SynthSeg+ enabled volumetric analysis. Linear mixed‐effects models examined impact of brain lesions, disease course, MOG‐Ab serostatus and age at onset on brain volumes and changes over time, along with clinical correlates.

**Results:**

Brain lesions were present in 71/109 patients, who were younger and more likely to present with acute disseminated encephalomyelitis. At onset, 79% showed reduced brain growth, particularly those with brain lesions. Over time, 46% developed atrophy, associated with lesion presence and relapsing disease.

All patients exhibited cortical and deep GM growth reduction at onset, with brain lesions driving progressive atrophy. Brian lesion complete resolution mitigated atrophy in the left supramarginal and right inferior parietal gyri. Relapsing disease was linked to greater GM atrophy in the frontal, temporal and parietal lobes. Persistent MOG‐Ab positivity correlated with GM atrophy in the cingulate and entorhinal cortices and temporal pole. Disability progression was linked to deep GM, temporal pole and lateral orbitofrontal atrophy, while learning difficulties were associated with lateral occipital and parietal atrophy.

**Interpretation:**

Brain lesions at onset and their persistence, relapsing disease and MOG‐Ab positivity are key risk factors for GM atrophy and clinical impairment in paediatric MOGAD.

## 1 Introduction

Myelin oligodendrocyte glycoprotein antibody (MOG‐Ab)–associated disease (MOGAD) is a demyelinating disease distinct from multiple sclerosis (MS) and aquaporin‐4 antibody neuromyelitis optica spectrum disorder (NMOSD) [1, 2, 3]. MOG‐Ab can be found in about one‐third of all cases of acquired demyelinating syndromes in children [4], and detectable in 50% of cases presenting with optic neuritis or acute disseminated encephalomyelitis (ADEM) [1, 5]. MOGAD generally follows a monophasic course in most individuals, although a relapsing course has been reported with frequencies from 17% to 38% in various studies [6, 7, 8]. Overall, a good and complete recovery from attacks has been described in paediatric MOGAD patients [9] without evidence of disability accrual independent from relapses [10].

In recent years, it has been demonstrated that not only MS but also acute demyelinating syndromes negatively affect brain developmental trajectories [11]. Moreover, a recent study showed that MOGAD onset during childhood causes negative deviations from the expected growth trajectory of deep grey matter structures, with accelerated changes occurring in the months immediately following an acute attack [12]. Studies conducted in adults with MOGAD suggested that the presence and the location of lesions in these patients affect the deep and cortical grey matter atrophy pattern [13, 14].

In paediatric MOGAD patients, brain lesions are dynamic both during an attack and following the acute presentation [15]. Among children with initially abnormal brain MRI, 83% will have at least one lesion resolved by the first follow‐up scan, and half will have a normal MRI after 1 year [16]. Moreover, compared to adults, children exhibit greater resilience against demyelinating attacks [17, 18], being provided with higher myelin repair capabilities [19, 20].

We evaluated whole brain and regional grey matter volume trajectories in a cohort of children with MOGAD to provide insights into the mechanisms underlying impaired brain growth and atrophy. In details, we compared deviations from age and sex‐expected brain and regional grey matter volumes at disease onset and over time between (1) children with and without brain MRI lesions at presentation, (2) children with new or persisting brain MRI lesions versus those whose lesion has completely resolved, (3) monophasic versus relapsing disease and (4) persistent MOG‐antibody positivity versus seroconversion. Furthermore, we aimed to test the effect of age at disease onset on whole brain and regional grey matter volume trajectories and the association of these trajectories with long‐term clinical disability, visual acuity and learning difficulties.

## 2 Methods

### 2.1 Subjects

This was a retrospective observational study using routine clinical care data. Paediatric patients were enrolled at two tertiary referral centres for autoimmune demyelinating disorders, Great Ormond Street Hospital (GOSH) for Children NHS Trust and Evelina London Children Hospital (London, United Kingdom). Our inclusion criteria were: (1) diagnosis of MOGAD according to the 2023 International MOGAD criteria [21]; (2) first clinical attack occurring before age of 18 years; (3) brain MRI obtained within 4 weeks of the first attack nadir (which was considered MRI at disease onset); (4) one or more follow‐up MRI performed at least 6 weeks after the disease onset MRI [patients had to be off steroids, (in UK, paediatric patients receive intravenous methylprednisolone followed by a tapering course of prednisone over 4–6 weeks) [22]]; (5) at all‐time points, MRI protocol included T1‐weighted images for volumetric analysis, T2‐weighted and/or fluid attenuated inversion recovery (FLAIR) for lesion assessment; (6) absence of other neurological or psychiatric diseases.

Clinical data including demographics, clinical findings (Expanded Disability Status Scale [EDSS], visual acuity measurement and learning difficulties [reported as school difficulties during clinical visits]), first and subsequent relapse characteristics, treatment information, neuroimaging reports and laboratory results were retrospectively collected from electronic medical records.

Patients were categorised into those with and without brain lesions. Patients with brain lesions on the initial scan, were further classified into: (i) patients with new or persisting lesions and (ii) patients with complete resolution of all pre‐existing lesions. To test the role of MOG‐Ab seroconversion the analysis was restricted to patients tested for MOG‐Ab within 6 months from the last available MRI scan. As MOG‐Ab testing was conducted in a clinical setting using either a fixed or live cell‐based assay (CBA), we considered only the serum status (positive vs. negative) and did not take titres into account.

To overcome the difficulty of obtaining longitudinal MRI scans from paediatric healthy controls, we selected a group of 317 paediatric healthy children from an NIH‐funded MRI Study of Normal Brain Development (NIH HC) with longitudinal MRI assessments for brain volume quantification (median follow‐up: 3.6, range = 0.9, 5.4 years) [23]. The data from the NIH‐funded MRI Study of Normal Brain Development were downloaded in August 2023. MRI data from all available healthy participants were initially downloaded, then an imaging quality check for motion and other artefacts was performed. From the 317 NIH HC considered suitable for the analysis, 163 had two scans (median time from first to second scan was 2 years between 0.9 and 4.3 years and 154 had 3 scans, median time from first to third was 3.9 years, between 2.0 and 5.4 years).

Additional details regarding the MRI acquisition protocol are available in Supporting Information S1 and in Table S1.

### 2.2 MRI Analysis

Within‐subject serial longitudinal registration in SPM12 was employed to align the available 3D T1‐weighted images [24]. For each subject, this procedure generated a subject‐specific mid‐point average template. Subsequently, all available 3D T1‐weighted images for each subject were aligned to this template. The 3D T1‐weighted images registered to the subject‐specific mid‐point average template were segmented using SynthSeg^+^ (https://surfer.nmr.mgh.harvard.edu/fswiki/SynthSeg) [25, 26]. By applying this segmentation technique, we obtained cortical grey matter segmented according to Desikan‐Killiany atlas [27] and deep grey matter as described by Fischl et al. [28]. All segmentations were visually inspected by an experienced observer (EDM). Global and regional grey matter volumes were normalised by dividing them by the total intracranial volume.

### 2.3 Z‐Scores Computation

To estimate the deviation from sex‐ and age‐expected whole brain and grey matter maturation trajectories in paediatric MOGAD patients, we calculated z‐scores for whole brain and grey matter regions at each timepoint, as per methodologies used in previous studies (details are available in Supporting Information S1) [11, 29].

### 2.4 Statistical Analysis

Patients were categorised into the following groups: (i) with versus without brain lesions at first presentation; (ii) with complete lesion resolution versus persistently abnormal brain MRI scans at follow‐up; (iii) with a monophasic versus relapsing disease course and (iv) with persistent MOG antibody positivity versus seroconversion to negative antibody status. Between‐group comparisons of demographic, clinical and structural MRI parameters at baseline were conducted using Pearson's chi‐square test, Mann–Whitney *U*‐test, two‐sample t‐test and linear models or linear mixed‐effects models, as appropriate.

At individual subject level we assessed reduced brain growth (defined as negative [*z*‐score < −1.5, a widely used threshold in neurodevelopmental and growths studies [30]] deviation from age‐ and sex‐specific developmental trajectories) and atrophy (defined as a progressively greater negative deviation from age‐ and sex‐specific brain developmental trajectories over time).

In the groups described above, we also assessed reduced growth and atrophy in individual grey matter regions. Using linear mixed‐effects models, we examined the influence of age at disease onset and MOG antibody seroconversion on z‐score trajectories over time. Finally, employing the same statistical approach, we evaluated how changes in grey matter volume over time could explain annualised changes in EDSS, visual acuity at follow‐up visits and learning difficulties.

Statistical analysis has been performed by using R version 4.3.2. Within each model, analyses are tested *p* < 0.05, false discovery rate (FDR) corrected for multiple comparisons (p values reported are FDR corrected).

### 2.5 Standard Protocol Approvals, Registrations and Patient Consents

This study was approved by Great Ormond Street Hospital Research and Development Department (reference: 16NC10). Any data not published within the article will be shared on request from any senior investigator.

## 3 Results

### 3.1 Demographic, Clinical and Conventional MRI Features

A total of 109 paediatric MOGAD patients fulfilled the inclusion criteria. Spinal cord lesions were seen in 12/71 of children with brain lesions and 3/38 of children without. Patients with brain lesions were younger (6.2 ± 3.7 vs. 8.7 ± 3.1; *p* < 0.001), more commonly presented with ADEM (64/71 vs. 4/38; *p* < 0.001), and less frequently with ON (6/71 vs. 30/38; *p* < 0.001) than patients without brain lesions. All the remaining demographic and clinical characteristics did not differ between paediatric MOGAD patients with and without brain lesions (Table 1).

**TABLE 1:** Summarises clinical features of paediatric MOGAD patients as a whole and grouped according to the presence of brain lesions at disease onset and to the degree of lesion resolution after the first attack.

Variable	All paediatric MOGAD patients	Paediatric MOGAD patients with brain MRI lesions	Paediatric MOGAD patients without brain MRI lesions	*p* (patients with versus those without brain lesions)
	*N* = 109	*N* = 71	*N* = 38	
*Baseline*				
Mean age at onset (SD)	7.0 (3.8)	6.2 (3.7)	8.7 (3.1)	< 0.001
Sex [M/F]	45/64	31/40	14/24	0.76
Type of onset (*n*, %)				
Brain, brainstem, or cerebral syndrome (including ADEM; cerebral monofocal or polyfocal deficits; brainstem or cerebellar deficits; cortical encephalitis)	69 (63%)	64 (90%)	5 (13%)	< 0.001
ON	36 (33%)	6 (8%)	30 (79%)	< 0.001
ON‐TM	2 (2%)	1 (2%)	1 (3%)	0.32
TM	2 (2%)	0 (0%)	2 (5%)	0.51
Median EDSS (IQR)	1.0 (0.0–1.0)	1.0 (0.0–1.5)	0.5 (0.0–1.0)	0.12
Acute treatment^a^ Steroids/IVIG/PLEX/None	75/11/9/21	44/10/8/16	31/1/1/5	0.11
*Follow‐up time*				
Median follow‐up duration (IQR) [years]	1.6 (0.5–3.9)	1.5 (0.5–4.1)	1.7 (0.6–3.5)	0.23
Relapsing (*n*, %)	50 (43%)	30 (40%)	20 (52%)	0.16
Median number of follow‐up scans (range)	2 (1–11)	2 (1–10)	2 (1–11)	0.09
MOG‐Ab seroconversion [Negative/Positive]	18/69	12/42	6/27	0.85
Median time to MOG‐Ab seroconversion (IQR)	3.3 (0.4, 6.6)	0.9 (0.3, 6.6)	4.5 (3.7, 6.1)	0.38
Maintenance immunomodulation [yes/no]	27/90	20/59	7/31	0.63
Type of manteinance immunomodulation (MMF/AZA/RTX/MMF + IVIG)	11/6/1/9	8/5/1/6	3/1/0/3	0.99
EDSS annualised change (SD)	0.1 (0.6)	0.2 (0.7)	0.1 (0.2)	0.45
EDSS at last follow‐up	1.0 (0.0–1.5)	1.0 (0.0–1.5)	0.5 (0.0–1.0)	0.12
Mean last Logmar score (SD) [left/right]	0.6 (0.4) 0.6 (0.5)	0.7 (0.4) 0.6 (0.5)	0.5 (0.4) 0.5 (0.5)	0.45 0.33
Learning difficulties [yes/no]	15/82	14/59	1/23	0.15

Abbreviations: Ab = antibodies, IQR = interquartile range, IVIG = intravenous immunoglobulin, MOGAD = myelin oligodendrocyte glycoprotein associated disease, PLEX = plasma exchange, SD = standard deviation.

^a^ There are patients assuming more than one treatment for the acute attack.

Over a median follow‐up of 1.6 years [interquartile range (IQR) 0.5–3.9, median number of scans per patient 2, IQR: 2–11] no differences in clinical outcomes were seen between patients with or without brain lesions (Table 1). Patients without brain lesions at disease onset continued to be lesion‐free at the last follow‐up (median follow‐up duration = 1.7 years, IQR = 0.6–3.5 years). In patients with brain lesions (median follow‐up duration = 1.5, IQR = 0.5–4.1 years), complete lesion resolution was seen in 21, 36 had persisting lesions, 9 new symptomatic lesions (associated with a clinical relapse) and 5 asymptomatic lesions. Of the 21 patients with complete lesion resolution only one patient had a relapse (involving the spinal cord).

### 3.2 Reduced Brain Growth (at Disease Onset) and Atrophy Over Time

At disease onset, reduced brain growth was observed in 86/109 (79%) patients. Patients with brain lesions at onset were more likely to experience reduced brain growth compared to those without lesions (62/71 vs. 24/38; *p* = 0.006). No differences in brain growth were found between patients whose lesions resolved and those with persisting or new lesions, nor between patients with relapsing and monophasic disease courses.

Over the follow‐up period, 50/109 (46%) of patients developed brain atrophy. This was associated with the presence of brain lesions at disease onset (38/71 vs. 12/38; *p* = 0.043) and with the persistence or development of new lesions compared to lesion resolution (31/50 vs. 7/21; *p* = 0.038). Patients with a relapsing disease course were more likely to develop brain atrophy than those with a monophasic course (30/46 vs. 20/63; *p* < 0.001). This difference was only observed in patients with brain lesions at onset (23/26 vs. 15/45; *p* < 0.001) but not in those without.

**FIGURE 1:**
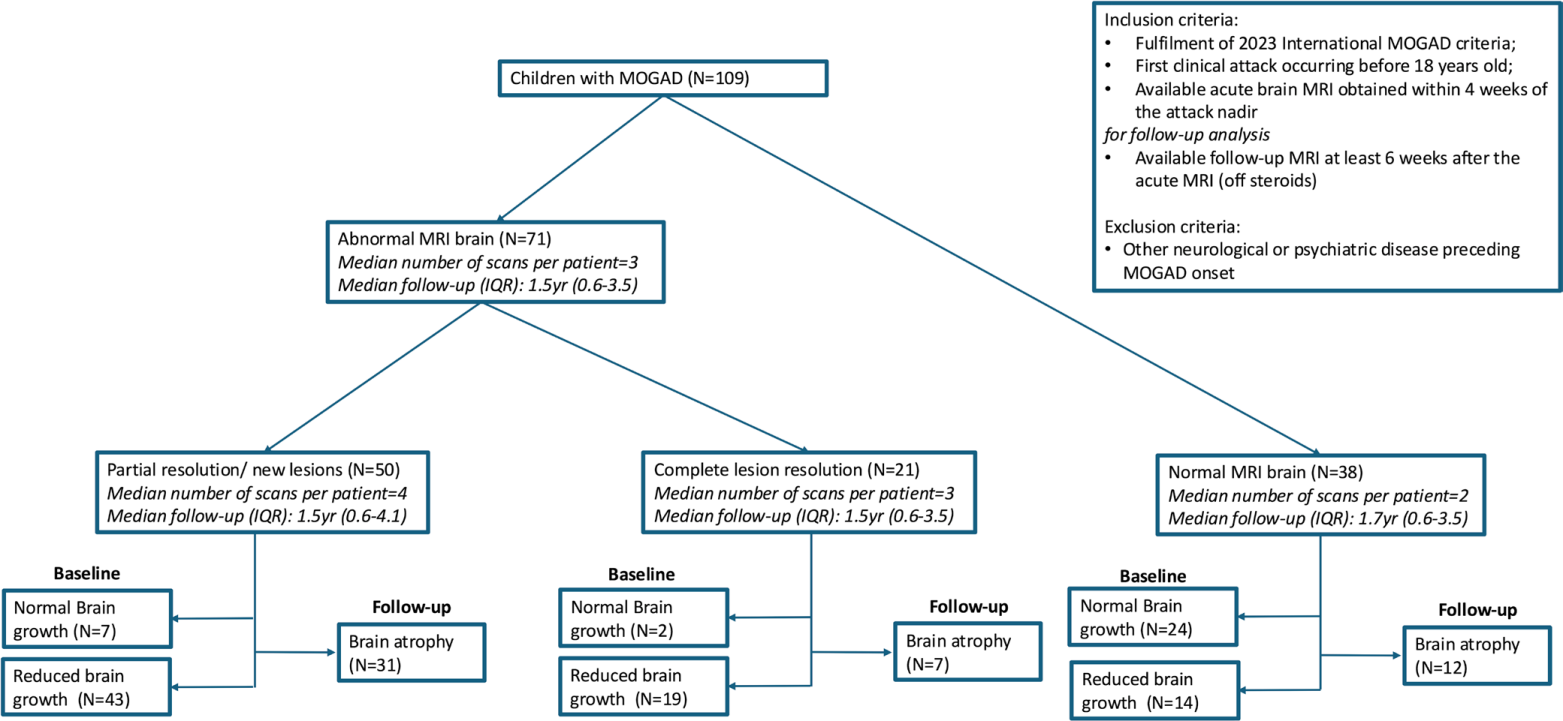
Study flow‐chart and prevalence of atrophy. Summarises the study flow‐chart and reports the prevalence of reduced brain‐growth and atrophy in paediatric MOGAD patients grouped by their lesion status.

**FIGURE 2:**
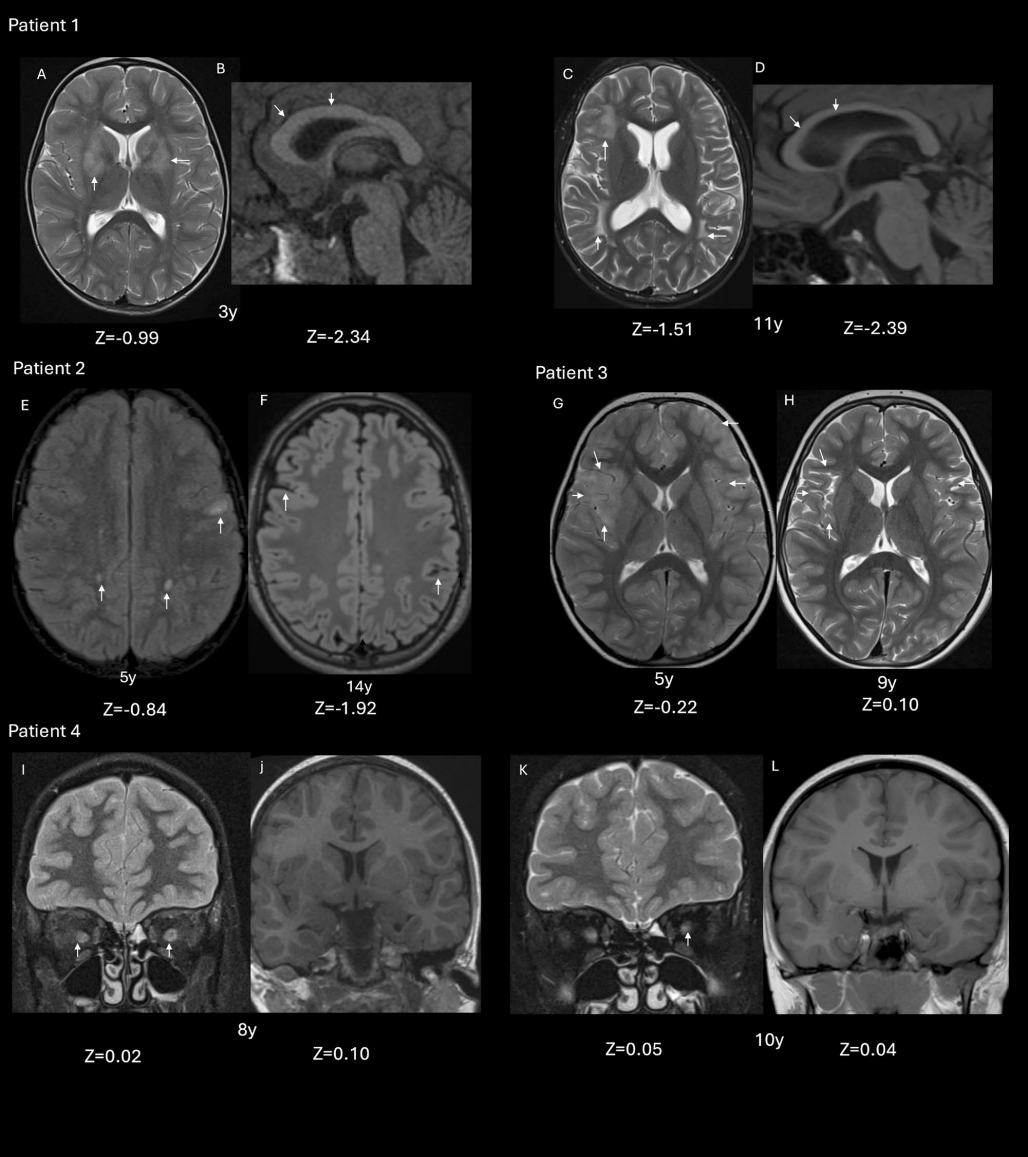
Iconographic representation of paediatric MOGAD patients. Patient 1: at the onset of disease, 3 years of age: axial T2 weighted image at the level of the basal ganglia (A) shows hyperintense lesions involving the caudate and putamina bilaterally (arrows, A). The overall brain volume is preserved with no sulcal or ventricular dilatation. Note the normal appearances of the corpus callosum (arrows, B). Follow‐up imaging, 11 years of age: Axial T2 weighted image at the level of the basal ganglia shows that the caudate and putaminal lesions have now resolved. There are, however, new white matter lesions in the frontal and temporoparietal white matter (arrows, C). Note diffuse volume loss evidenced by the prominence of the ventricles and sulcal spaces (C), and white matter volume loss represented by callosal thinning on the sagittal T1 weighted image (D). Patient 2: At onset of disease, 5 years of age: Axial FLAIR sequence shows multiple white matter lesions involving the frontal and parietal white matter (arrows, E). The overall brain volume is preserved with no sulcal dilatation. Follow up imaging, 14 years of age: Axial FLAIR sequence shows resolution of the white matter lesions. There is now an overall decrease in brain volume evidenced by prominent sulcal spaces in (arrows F). Patient 3: At onset of disease, 5 years of age. Axial T2 weighted image shows swelling and hyperintensity involving the bilateral fronto‐insular and opercular cortices (right > left) (arrows, G). On follow up imaging at 9 years of age, there is no whole brain volume loss with resolution of previously seen lesions (arrows, H). Patient 4: Coronal saturated T2 weighted image shows bilateral optic neuritis (arrows, I). Coronal T1 weighted image shows preserved brain volume. Brain volume is preserved (J). Follow up imaging at 10 years of age shows residual signal change within the optic nerves with atrophy (arrow, K). No brain volume loss is evident (L).

Figure 1 reports the study flowchart, illustrating the prevalence of reduced brain growth and atrophy, while Figure 2 provides MRI examples representing patients from each group.

### 3.3 Regional Grey Matter Patterns of Deviations From Age‐ and Sex‐Expected Growth

#### 3.3.1 Paediatric MOGAD Patients With Versus Without Brain MRI Lesions

Considering the regional grey matter patterns of deviations from age‐ and sex‐expected developmental trajectories, both patients with and without brain lesions exhibited negative deviations in regions of the frontal, parietal, temporal and occipital lobes, as well as in deep grey matter (intercept from −1.71 to −0.56; FDR‐corrected *p* < 0.001 to 0.048). In contrast, positive deviations were observed in the cingulate cortex and regions within the medial occipital and temporal lobes (intercept from 0.56 to 4.51; FDR‐corrected *p* < 0.001 to 0.049).

Compared to patients without brain lesions, those with brain lesions exhibited higher z‐scores in the bilateral cingulate cortex, medial occipital and temporal lobes, and the frontal pole (*β* from 0.52 to 0.77; FDR‐corrected *p* < 0.001 to 0.047) but lower z‐scores in the left inferior parietal lobule (*β* = −0.66; FDR‐corrected *p* = 0.022).

**FIGURE 3:**
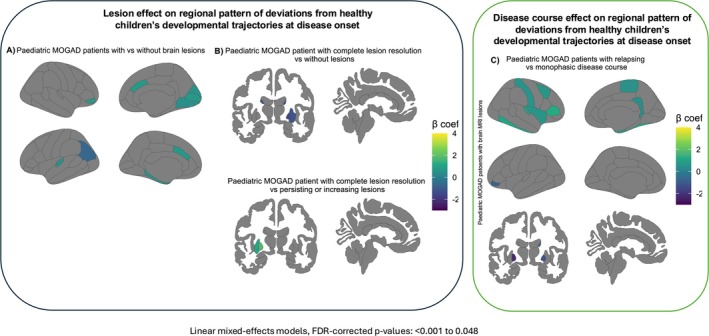
Regional grey matter deviations from developmental trajectories at disease onset. Significant differences in deviations from healthy children's developmental trajectories in: (A) paediatric MOGAD patients with versus without brain lesions; (B) paediatric MOGAD patients with complete lesion resolution versus those with persisting or new lesions an versus those without brain MRI lesions (C) paediatric MOGAD patients with relapsing versus monophasic disease course. FDR = false discovery rate, MOGAD = myelin oligodendrocyte glycoprotein antibody‐associated disease.

Figure S1 and Table S2 detail deviations from healthy development in paediatric MOGAD patients with and without brain lesions, including group comparisons. Figure 3A summarises key differences between these patient groups.

#### 3.3.2 Paediatric MOGAD Patients With Complete Lesion Resolution Versus Patients With Persisting or Increasing Lesions and Those Without Brain MRI Lesions

At disease onset, patients with subsequent complete brain lesion resolution, exhibited negative deviations in regions of the frontal, parietal, temporal and occipital lobes, as well as in deep grey matter (intercepts from −1.64 to −0.72; FDR‐corrected *p* from < 0.001 to 0.042). In contrast, positive deviations were observed in brain regions within the cingulate cortex and medial occipital and temporal lobes (intercepts from 0.90 to 4.42; FDR‐corrected *p* from < 0.001 to 0.019).

When compared to patients with persisting or new brain lesions, those with complete brain lesion resolution exhibited higher z‐scores in left caudate nucleus and putamen (*β* from 1.17 to 1.81; FDR‐corrected *p* 0.001 to 0.049). Conversely, they displayed lower z‐scores in deep grey matter regions (*β* from −1.56 to −1.04; FDR‐corrected *p* from 0.031 to 0.046), when compared to paediatric MOGAD patients without brain lesions (*n* = 38).

Figure S2 and Table S3 detail deviations from healthy development in paediatric MOGAD patients with resolving versus persisting/increasing or without brain lesions. Figure 3B summarises key differences among these groups.

#### 3.3.3 Paediatric MOGAD Patients With Relapsing Versus Monophasic Disease Course

Among patients with brain lesions, those with a monophasic disease course, compared to those with a relapsing course, demonstrated higher z‐scores in the right middle frontal gyrus, fusiform gyrus, inferior temporal gyrus, postcentral gyrus, transverse temporal gyrus, entorhinal and isthmus cingulate cortices, insula and paracentral lobule (*β* from 0.55 to 1.18; FDR‐corrected *p* < 0.001 to 0.047). They also exhibited lower z‐scores in the bilateral pallidum, right caudate nucleus and pars orbitalis of the left frontal gyrus (*β* from −2.31 to −0.75; FDR‐corrected *p* < 0.001 to 0.047).

No differences in z‐scores at disease onset were observed among patients without brain lesions when comparing those with monophasic and relapsing disease courses.

Figure S3 and Table S4 detail developmental deviations in paediatric MOGAD patients with relapsing or monophasic courses, stratified by brain lesions at onset. Figure 3C summarises key differences.

### 3.4 Regional Grey Matter Atrophy Patterns (Deviations From Age‐ and Sex‐Expected Growth Over Time)

#### 3.4.1 Paediatric MOGAD Patients With and Without Brain Lesions

Over time, patients with brain lesions exhibited progressively reduced z‐scores across the bilateral frontal, temporal, parietal and occipital lobes, as well as in the deep grey matter and cingulate cortex (*β* from −0.44 to −0.10; FDR‐corrected *p* < 0.001 to 0.042). In contrast, patients without brain lesions showed no significant changes over time in any grey matter region.

Notably, patients with brain lesions experienced further reductions in z‐scores over time, particularly in the right thalamus and regions within the bilateral frontal, parietal, temporal and occipital lobes, compared to those without brain lesions (β from −0.44 to −0.10; FDR‐corrected *p* < 0.001 to 0.043).

**FIGURE 4:**
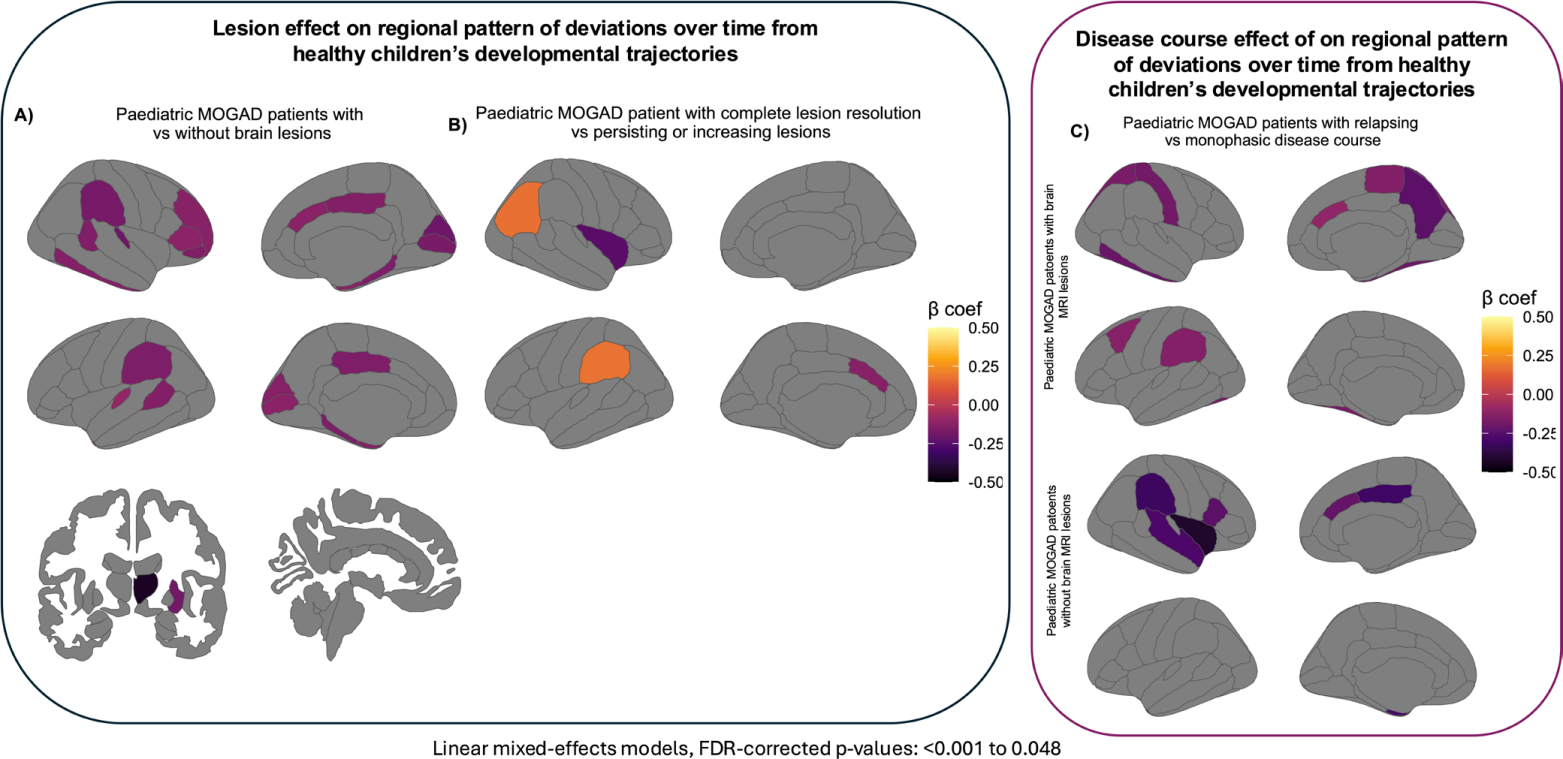
Regional grey matter deviations over time in paediatric MOGAD patients with and without brain lesions. Significant differences in deviations over time from healthy children's developmental trajectories in: (A) paediatric MOGAD patients with versus without brain lesions; (B) paediatric MOGAD patients with complete lesion resolution versus those with persisting or new lesions and versus those without brain MRI lesions; (C) paediatric MOGAD patients with relapsing versus monophasic disease course. FDR = false discovery rate, MOGAD = myelin oligodendrocyte glycoprotein antibody‐associated disease.

Figure S4 and Table S5 detail changes in developmental deviations over time in paediatric MOGAD patients with and without brain MRI lesions, including group comparisons. Figure 4A summarises key differences.

#### 3.4.2 Paediatric MOGAD Patients With Complete Lesion Resolution Versus Patients Without Brain MRI Lesions and Those With Persisting or New Lesions

Compared to patients with persisting or new lesions, those with complete lesion resolution demonstrated a greater reduction in z‐scores over time in the left anterior cingulate cortex and right insula (*β* from −0.25 to −0.13; FDR‐corrected *p* = 0.047 to 0.048) and a smaller reduction in the left supramarginal and right inferior parietal gyri (*β* from 0.17 to 0.18; FDR‐corrected *p* = 0.048 to 0.049). No differences were observed when comparing these patients to patients without brain lesions.

Figure S5 and Table S6 detail longitudinal changes in developmental deviations in paediatric MOGAD patients with resolving versus persisting/increasing, or without brain lesions. Figure 4B summarises key differences.

#### 3.4.3 Paediatric MOGAD Patients With Relapsing Versus Monophasic Disease Course

Among patients with brain lesions, those with a relapsing disease course exhibited a greater reduction in z‐scores over time compared to those with a monophasic course in the bilateral middle frontal and fusiform gyri, left supramarginal gyrus, right inferior temporal gyrus, postcentral gyrus, superior parietal gyrus, paracentral lobule and precuneus (*β* from −0.24 to −0.11; FDR‐corrected *p* = 0.002 to 0.044).

Among patients without brain lesions, those with a relapsing disease course exhibited greater reductions in z‐scores over time compared to those with a monophasic course in the left entorhinal cortex and temporal pole, right cingulate cortex, superior temporal and supramarginal gyri, insula and pars opercularis of the frontal gyrus (*β* from −0.42 to −0.29; FDR‐corrected *p* = 0.028 to 0.042).

Figure S6 and Table S7 detail longitudinal developmental deviations in paediatric MOGAD patients with relapsing or monophasic courses, stratified by brain MRI lesions at onset. Figure 4C summarises key differences.

### 3.5 MOG‐Ab Serum Status Effect on Grey Matter Z‐Scores

Compared to seroconverting patients, those with persistent MOG‐Ab showed no differences at disease onset but reduced z‐scores over time (median follow up time = 1.6 years, IQR: 0.5–3.9) in the bilateral cingulate cortex, left entorhinal cortex, temporal pole and lingual gyrus, as well as in the right post‐central gyrus. Table 2 summarises the differences in grey matter z‐scores over time for patients with persistent MOG‐Ab compared to seroconverting patients.

**TABLE 2:** Summarises the effect of persistent MOG‐Ab positivity on grey matter z‐scores over time.

	Brain region	*β* coef	SE	*p*
MOG‐Ab serostatus	Left entorhinal cortex	−0.13	0.04	< 0.001
	Left isthmus cingulate cortex	−0.16	0.05	0.010
	Left lingual gyrus	−0.10	0.03	0.019
	Left temporal pole	−0.13	0.03	< 0.001
	Right caudal anterior cingulate cortex	−0.10	0.04	0.008
	Right isthmus cingulate cortex	−0.10	0.04	0.036
	Right postcentral gyrus	−0.12	0.05	0.046
	Right posterior cingulate cortex	−0.14	0.03	< 0.001

Abbreviations: Coef = coefficient, SE = standard error.

### 3.6 Age at Onset Effect on Grey Matter Z‐Scores

Higher age at disease onset was associated with lower z‐score in left anterior cingulate cortex, lingual, parahippocampal and transverse temporal gyrus and right pericalcarine cortex, while it was associated with a slower reduction of z‐scores over time in left parahippocampal, post‐central, transverse temporal and supramarginal gyrus and in right cuneus, inferior parietal gyrus and pars orbitalis of inferior frontal gyrus. Table 3 summarises age at onset effect on deviations form healthy children's grey matter developmental trajectories.

**TABLE 3:** Summarises age at onset effect on grey matter z‐score in paediatric MOGAD patients at disease onset and over the follow‐up time.

At disease onset				Over time			
Brain region	*β* coef	SE	*p*	Brain region	*β* coef	SE	*p*
Left caudal anterior cingulate cortex	−0.13	0.02	< 0.001	Left parahippocampal gyrus	0.02	0.01	0.010
Left lingual gyrus	0.10	0.02	< 0.001	Left postcentral gyrus	0.03	0.01	0.023
Left parahippocampal gyrus	−0.13	0.02	< 0.001	Left supramarginal gyrus	0.04	0.01	< 0.001
Left transverse temporal gyrus	−0.15	0.02	< 0.001	Left transverse temporal gyrus	0.02	0.01	0.010
Right pericalcarine cortex	−0.12	0.02	< 0.001	Right cuneus	0.03	0.01	0.003
				Right inferiorparietal gyrus	0.02	0.01	0.016
				Right pars orbitalis of inferior frontal gyrus	0.02	0.01	0.004

Abbreviations: Coef = coefficient, SE = standard error.

### 3.7 Association With Clinical Measures

No associations were observed between z‐scores at disease onset and EDSS changes over time. EDSS increase over time was associated with reduced z‐scores over time in bilateral thalamus, left hippocampus, temporal pole and lateral orbitofrontal cortex, right accumbens, anterior cingulate cortex, fusiform, inferior temporal and lingual gyri. Patients with learning difficulties (15 out of 82) experienced greater reductions in z‐scores over time in the bilateral lateral occipital gyri, left superior parietal gyrus and right inferior parietal gyrus. Table 4 summarises the association between z‐scores changes over time with annualised EDSS changes and learning difficulties. No associations were observed between z‐scores at disease onset and over time with visual acuity at the last follow‐up.

**TABLE 4:** Summarises associations between baseline deviations from age‐ and sex‐expected trajectories and EDSS annualised changes.

	Brain regions	*β* coef	SE	*p*
EDSS	Left thalamus	−0.13	0.04	< 0.001
	Left hippocampus	−0.06	0.02	0.019
	Left ventral dienecephalon	−0.08	0.03	< 0.001
	Right thalamus	−0.13	0.04	< 0.001
	Right nucleus accumbens	−0.06	0.02	0.032
	Left lateral orbitofrontal cortex	−0.04	0.02	0.049
	Left temporal pole	−0.02	0.01	0.046
	Right caudal anterior cingulate cortex	−0.03	0.01	0.027
	Right fusiform gyrus	−0.04	0.01	0.042
	Right inferior temporal gyrus	−0.04	0.01	0.023
	Right lingual gyrus	−0.03	0.01	0.048
Learning difficulties	Left lateral occipital gyrus	−0.17	0.05	< 0.001
	Left superior parietal gyrus	−0.10	0.04	0.044
	Right inferior parietal gyrus	−0.12	0.04	0.021
	Right lateral occipital gyrus	−0.12	0.04	< 0.001

Abbreviations: Coef = coefficient, EDSS = Expanded Disability Status Scale, SE = standard error.

## 4 Discussion

In a large cohort of paediatric patients with MOGAD, we observed reduced brain growth in 80% of patients, with brain atrophy observed in 46%. Our results highlight that persistence of lesions over time, along with repeated demyelinating attacks, is a key factor in determining brain atrophy. Patients with persisting or new brain lesions were twice as likely to develop brain atrophy compared to those whose brain lesions completely resolved (62% vs. 31%). As both lesion resolution following an attack [16] and relapse prevention [31] are influenced by the timing and choice of treatment, our results highlight that, beyond attack recovery and prevention, optimal treatment has long‐term implications.

As MOGAD lesions have been shown to exhibit less microstructural disruption than MS lesions [32], and are more likely to resolve over time [16], we can speculate that only the more disruptive lesions contribute to Wallerian degeneration‐mediated brain atrophy. Furthermore, the association between relapsing disease and both more frequent failures in brain growth and increased atrophy, confirms not only the concept of ‘attack‐mediated brain insult’, with more severe volume loss in the months following an attack [12], but also suggests that differences between relapsing and monophasic phenotypes may exist from the disease onset. Considered together, these findings allow us to hypothesise that individual resilience against inflammatory damage can be the main determinant of disease‐related damage.

We also investigated regional grey matter volume changes at disease onset and over time. The regions exhibiting increased grey matter volumes, included the cingulate cortex and medial temporal lobe, regions typically involved in limbic encephalitis [33, 34]. Data from animal and human studies suggests that this increased volume may result from the entry of immune cells into the cerebrospinal fluid (CSF) via meningeal lymphatic vessels and blood–brain barrier disruption, which was shown to be key factors in the pathogenesis of autoimmune encephalitis [35, 36, 37, 38]. Interestingly, a more widespread pattern of increased grey matter volume was observed at disease onset in patients with brain lesions compared to those without and in patients with relapsing compared to those with monophasic disease course. This suggests that more severe disease may be linked to increased blood–brain barrier permeability, in line with studies showing that such permeability predicts prognosis and treatment response in autoimmune encephalitis [33].

Impaired maturation at disease onset was observed in both deep grey matter and cortical grey matter regions across the entire population studied, with minimal differences between groups. This finding highlights the impact of individual attacks on brain developmental trajectories [11, 12], suggesting that children who experience acquired CNS demyelination have increased vulnerability, or less resilience, to the negative effects of an immunological attack on brain growth, regardless of lesions [11]. Indeed, while reduced brain growth in paediatric MS patients could also be attributed to the effects of lesions during a variable‐length subclinical phase before the disease becomes manifest, this is unlikely in MOGAD, which typically has a more dramatic onset [39].

The widespread atrophy, across multiple brain areas, observed in patients with brain lesions, was also reported in paediatric MS [29] and is likely a result of both retrograde and anterograde degeneration. Interestingly, patients with complete brain lesion resolution showed significant reduction in grey matter volume over time only in those regions exhibiting higher volume at disease onset. This suggests that the decrease in z‐scores in these regions may represent the resolution of the acute inflammatory condition or the restoration of blood–brain barrier integrity rather than atrophy accrual. Moreover, we did not observe differences in grey matter volume changes when comparing these patients to those without brain lesions. However, higher z‐scores over time were observed in the supramarginal and inferior parietal gyri when comparing these patients to those with persisting or increasing lesions. These findings highlight the potential for recovery or greater resilience in late‐maturing regions [40] and suggest that lesion resolution is a positive predictive factor against the development of atrophy.

While several studies have demonstrated that persistent MOG‐Ab status, is associated with a relapsing disease course [41, 42, 43, 44], we also observed that those patients with sustained MOG‐Ab positivity experience more pronounced brain volume loss in regions susceptible to increased blood–brain barrier permeability during attacks. While the heightened risk of relapse linked to persistent MOG‐Ab positivity [41, 42, 43, 44] is likely a key factor in the development of brain atrophy, we cannot exclude the direct role of MOG‐Ab in ongoing low‐level inflammation outside of clinical attacks.

As expected, younger children were more likely to develop brain atrophy, aligning with previous reports indicating an increased likelihood of cognitive impairment in this age group [44].

Finally, we assessed the impact of volumetric changes on clinical outcomes, including clinical disability, visual acuity and learning ability. While we observed an association between EDSS increase and volume loss in key regions, such as the thalamus, known to be associated with clinical disability progression [29], no significant relationships were found between volumetric changes and visual acuity at follow‐up. It is worth noting that most of our patients experienced a complete recovery after optic neuritis, likely preventing us from assessing any association between this measure and volume loss. Learning disability was associated with volume loss in the parietal and occipital cortex. This finding aligns with evidence of abnormalities in the parieto‐occipital cortex mediating dyslexia [45].

This study has limitations. Healthy controls were not included to account for scanner variability, and only 65/109 paediatric MOGAD patients underwent MRI with the same scanner over the entire follow‐up duration; though multiple scanners make systematic error unlikely, with our validated segmentation technique ensuring reliable results across varying MRI contrast and resolution [25]. Most initial scans were obtained during acute attacks, with 70% receiving immunomodulatory treatment after the first MRI, raising concerns about pseudoatrophy [46]. However, as focused on grey matter, not whole‐brain or white matter volumes, the impact is likely minimal. We did not have the opportunity to specifically assess T1 hypointense lesions (‘black holes’), as only 3D T1‐weighted Fast Field Echo (FFE) sequences were available. Although FFE can depict T1 hypointensities, these are typically more reflective of transient changes such as oedema and provide limited additional value over conventional T2‐weighted imaging in identifying chronic tissue damage. An inherent limitation of the present study is its inability to disentangle the effects of acute and maintenance immunomodulatory treatment on atrophy development, as nearly all patients received acute treatment, while only a small proportion underwent long‐term therapy—likely those with a more aggressive disease course. Furthermore, although treatment‐related pseudoatrophy effects might have been better isolated in patients without new or persistent lesions, the limited number of untreated patients in this subgroup precluded a meaningful analysis. Due to the lack of systematic assessment, the impact of spinal cord and optic nerve lesions on grey matter atrophy was not evaluated. As children were tested for MOG‐Ab in a clinical setting, antibody titres were not measured, preventing a more detailed classification that distinguishes between increasing, decreasing and stable MOG‐Ab status. Lastly, learning disabilities were parent‐reported rather than assessed with a formal scale, possibly underestimating milder impairments. Finally, the limited follow‐up duration precluded an analysis of long‐term effects of brain lesions, MOG‐Ab and relapsing disease course on grey matter atrophy.

In conclusion, our study confirms that MOGAD impairs grey matter maturation and overall brain growth. Grey matter atrophy is primarily driven by persistent brain lesions, while patients with complete lesion resolution experience no additional volume loss. These findings suggest a therapeutic window for both acute attack management and relapse prevention.

## Author Contributions

E.D.M., O.C. and Y.H. contributed to the conception and design of the manuscript. E.D.M., O.C. and Y.H. contributed to the interpretation of studies included in the manuscript. E.D.M., R.N., M.E., C.H., M.L., T.R., A.B., K.M., A.S., S.S., D.C., F.B., A.E., O.C. and Y.H. contributed to drafting the text and preparing the figures.

## Conflicts of Interest

The authors declare no conflicts of interest.

## Supporting information


Data S1.

## Data Availability

Any data not published within the article will be shared on request from any senior investigator.
