# Supplementary material for: Understanding Mechanisms of Whole Brain and Regional Grey Matter Atrophy in Children With MOGAD
Source: Ann Clin Transl Neurol. 2025 Jul 2;12(10):1971–82. doi: 10.1002/acn3.70123 (PMC12516224; doi:10.1002/acn3.70123)
Supplement: Supplementary file 1 — Data S1. [file ACN3-12-1971-s001.docx]

**Supplementary Materials**

- **MRI acquisition**
- **Table 1.** MRI sequence acquisition parameters across the different MRI scanners.
- **Z-scores computation**
- **Figure 1.** Deviations from healthy children’s developmental trajectories at disease onset in paediatric MOGAD patients with and without brain lesions at disease onset, and the comparison between the two groups.
- **Table 2.** Deviations from healthy children’s developmental trajectories in paediatric MOGAD patients with and without brain MRI lesions at disease onset and the comparison between the two groups.
- **Figure 2.** Deviations from healthy children’s developmental trajectories at disease onset in paediatric MOGAD patients with complete brain lesion resolution, compared to those of patients with persisting or increasing lesions and those of patients without brain lesions.
- **Table 3.** Deviations from healthy children’s developmental trajectories at disease onset in paediatric MOGAD patients with complete brain lesion resolution, compared to those of patients with persisting or increasing lesions and those of patients without brain lesions.
- **Figure 3.** Deviations from healthy children’s developmental trajectories at disease onset in paediatric MOGAD patients with relapsing and monophasic disease course and the comparison between the two groups, grouping patients by the presence of brain lesions at disease onset.
- **Table 4.** Deviations from healthy children’s developmental trajectories at disease onset in paediatric MOGAD patients with relapsing and monophasic disease course and the comparison between the two groups, grouping patients by the presence of brain lesions at disease onset.
- **Figure 6.** Changes over time of deviations from healthy children’s developmental trajectories in paediatric MOGAD patients with and without brain lesions and the comparison between the two groups.
- **Table 5.** Changes over time of deviations from healthy children’s developmental trajectories in paediatric MOGAD patients with and without brain lesions and the comparison between the two groups.
- **Figure 5.** Changes over time of deviations from healthy children’s developmental trajectories in paediatric MOGAD patients with complete lesion resolution, compared to those of patients with persisting or increasing brain lesions and patients without brain lesions.
- **Table 5.** Changes over time of deviations from healthy children’s developmental trajectories in paediatric MOGAD patients with complete lesion resolution, compared to those of patients with persisting or increasing brain lesions.
- **Figure 6.** Changes over time of deviations from healthy children’s developmental trajectories at in paediatric MOGAD patients with relapsing and monophasic disease course and the comparison between the two groups, grouping patients by the presence of brain lesions at disease onset
- **Table 6.** Changes over time of deviations from healthy children’s developmental trajectories in paediatric MOGAD patients with monophasic and relapsing course at disease onset grouped according to the presence of brain MRI lesions at disease onset.

**MRI acquisition**

All available MRI were clinically acquired as part of the patient routine clinical care and reported by a paediatric neuroradiologist with expertise in demyelinating diseases. Patients were scanned on Philips, GE, or Siemens 1.5T scanners. The following brain MRI images were acquired:

**Table 1.** MRI sequence acquisition parameters across the different MRI scanners.

| Scanner | Sequence | TR (ms) | TE (ms) | ETL | Flip Angle (¬∞) | Inversion Time (ms) | Matrix Size | FOV (mm^2^) | Slices | Voxel Size (mm^2^) |
| --- | --- | --- | --- | --- | --- | --- | --- | --- | --- | --- |
| Philips 1.5T | **T2 FSE (2D)** | 4500-5500 | 95-120 | 14-16 | 90° | N/A | 256x256 | 230x230 | 40-50 | 1.0x1.0x3.0 |
|  | **FLAIR (2D)** | 6000-7000 | 120-250 | N/A | 110°-118° | 1650-2000 | 256x256 | 240x240 | 48 | 1.0x1.0x3.0 |
|  | **FLAIR (3D)** | 7000-8000 | 125-380 | N/A | 115°-120° | 1800-2200 | 240x240x160 | 250x250x160 | 160 | 1.0x1.0x1.0 |
|  | **3D-T1 FFE** | 1500-1700 | 2.30-3.40 | N/A | 8°-9° | N/A | 256x256 | 230x230 | 220 | 1.0x1.0x0.97 |
| GE 1.5T | **T2 FSE (2D)** | 4200-5300 | 92-125 | 13-15 | 90° | N/A | 240x240 | 225x225 | 40-50 | 1.0x1.0x3.0 |
|  | **FLAIR (2D)** | 6100-7200 | 110-260 | N/A | 108°-115° | 1700-2100 | 256x256 | 245x245 | 48 | 1.0x1.0x3.0 |
|  | **FLAIR (3D)** | 6900-7900 | 120-392 | N/A | 110°-118° | 1750-2150 | 256x256 | 250x250 | 160 | 1.0x1.0x1.0 |
|  | **3D-T1 FFE** | 1450-1600 | 2.27-3.43 | N/A | 8°-9° | N/A | 256x256 | 230x230 | 220 | 1.0x1.0x0.97 |
| Siemens 1.5T | **T2 FSE (2D)** | 4700-5600 | 98-125 | 15-17 | 90° | N/A | 250x250 | 235x235 | 40-50 | 1.0x1.0x3.0 |
|  | **FLAIR (2D)** | 6200-7300 | 115-270 | N/A | 110°-120° | 1750-2050 | 256x256 | 250x250 | 48 | 1.0x1.0x3.0 |
|  | **FLAIR (3D)** | 7100-8200 | 130-400 | N/A | 112°-120° | 1850-2250 | 240x240x160 | 260x260x160 | 160 | 1.0x1.0x1.0 |
|  | **3D-T1 FFE** | 1520-1680 | 2.35-3.45 | N/A | 8°-9° | N/A | 256x256 | 230x230 | 220 | 1.0x1.0x0.97 |

Abbreviations: TR=Repetition Time; TE=Echo Time; ETL=Echo Train Length; FOV=Field of View; FSE=Fast-Spin Echo; FLAIR=Fluid Attenuated Inversion Recovery; FFE=Fast-field Echo.

Scans of the NIH HC were obtained at six paediatric centers on GE and Siemens 1.5 Tesla scanners. The standardized MRI protocol included: a) DE TSE (TR/TE=3500/5-119 ms; ETL=8; flip angle=90°; matrix size=256×256; FOV=256×224 mm^2^; 2-mm-thick axial slices); b) whole brain 3D *T*_1_W RF-spoiled gradient echo sequence (TR=22-25 ms, TE=10-11 ms, flip angle=30°, FOV=256× (160-180) mm^2^, matrix=256×256, slice thickness=1.0 mm). Additional details on acquisition parameters and participants are described in Evans et al.^2^

**Z-scores computation**

To estimate age- and sex- specific brain and grey matter (GM) developmental trajectories the following linear mixed effects model was applied on the normalized whole brain and regional GM volumes of the healthy subjects:

$$V_{ij}= \beta_{0}+b_{i0}+\left( \beta_{1}+b_{i1} \right){Age}_{ij}+ \beta_{2}{Sex}_{i}+ \beta_{3}{Age}_{ij}*{Sex}_{i}+ \beta_{4}{Age}_{ij}^{2}+\varepsilon_{ij}$$

where *V_ij_* represent the volume of the whole brain or of a specific GM region for the subject *i* at the age *j*.

To estimate deviations from the expected maturation trajectories of the different brain regions in MOGAD patients, we calculated z-scores for whole brain and each GM region at every timepoint. As detailed in previous studies,^6, 8^ z-scores were computed by subtracting the mean and dividing by the standard deviation (SD), which was estimated from the variance-covariance matrix of the fixed effects and the residual variance of the random effects, using the age and sex for the specific participant.

To ensure the validity of Z-score computation, we assessed two key statistical assumptions: normality of residuals and homoscedasticity. Normality was evaluated through histograms, Q-Q plots, and Shapiro-Wilk tests, applied across different age groups (<5, 5–10, 10–15, >15 years). Homoscedasticity was tested using Levene’s test to determine whether variance remained stable across age groups, supplemented by visual inspection of residual plots.

Both normality of residuals and homoscedasticity assumptions were satisfied.

**Figure 1.** Summarizes deviations from healthy children’s developmental trajectories at disease onset in paediatric MOGAD patients with and without brain lesions at disease onset, and the comparison between the two groups.

**Deviation from age- and sex- expected maturational trajectories at disease onset in paediatric MOGAD patients with and without brain MRI lesions**

Intercept

Intercept

Linear mixed-effects models, FDR-corrected p-values <0.001 to 0.048

**Table 2.** Summarizes deviations from healthy children’s developmental trajectories in paediatric MOGAD patients with and without brain MRI lesions at disease onset and the comparison between the two groups.

| **Deviations from healthy children’s developmental trajectories in paediatric MOGAD patients with brain lesions** | | | |
| --- | --- | --- | --- |
| **Brain Region** | **Intercept** | **SE** | *******  ***p***  **values** |
| Left caudate nucleus | -0.61 | 0.13 | <0.001 |
| Left putamen | -0.98 | 0.15 | <0.001 |
| Left pallidum | -1.71 | 0.25 | <0.001 |
| Left nucleus accumbens | -0.77 | 0.16 | <0.001 |
| Right caudate nucleus | -0.58 | 0.14 | 0.023 |
| Right putamen | -0.71 | 0.13 | <0.001 |
| Right pallidum | -1.07 | 0.20 | <0.001 |
| Right nucleus accumbens | -0.71 | 0.16 | <0.001 |
| Left banks of superior temporal sulcus | -0.56 | 0.11 | <0.001 |
| Left caudal middle frontal gyrus | 0.72 | 0.11 | <0.001 |
| Left entorhinal cortex | 1.68 | 0.10 | <0.001 |
| Left inferior parietal lobule | -1.33 | 0.11 | <0.001 |
| Left inferior temporal gyrus | -0.80 | 0.11 | <0.001 |
| Left lingual gyrus | 0.93 | 0.09 | <0.001 |
| Left middle temporal gyrus | -1.18 | 0.11 | <0.001 |
| Left pars opercularis of inferior frontal gyrus | -0.75 | 0.09 | <0.001 |
| Left pars triangularis of inferior frontal gyrus | -1.03 | 0.10 | <0.001 |
| Left pericalcarine cortex | 3.38 | 0.16 | <0.001 |
| Left postcentral gyrus | -1.18 | 0.13 | <0.001 |
| Left posterior cingulate cortex | 4.41 | 0.21 | <0.001 |
| Left precentral gyrus | -1.13 | 0.13 | <0.001 |
| Left precuneus | -0.65 | 0.11 | <0.001 |
| Left rostral anterior cingulate cortex | -0.53 | 0.10 | <0.001 |
| Left rostral middle frontal gyrus | -1.49 | 0.12 | <0.001 |
| Left superior frontal gyrus | -0.66 | 0.14 | <0.001 |
| Left superior temporal gyrus | -1.13 | 0.13 | <0.001 |
| Left supramarginal | -1.02 | 0.10 | <0.001 |
| Left frontal pole | 4.51 | 0.25 | <0.001 |
| Left temporal pole | 0.79 | 0.11 | <0.001 |
| Right banks of superior temporal sulcus | 1.21 | 0.13 | <0.001 |
| Right caudal anterior cingulate cortex | 1.61 | 0.11 | <0.001 |
| Right caudal middle frontal gyrus | 1.78 | 0.12 | <0.001 |
| Right entorhinal cortex | 2.92 | 0.15 | <0.001 |
| Right fusiform gyrus | 2.89 | 0.18 | <0.001 |
| Right inferior temporal gyrus | 1.78 | 0.13 | <0.001 |
| Right isthmuscingulate | 0.85 | 0.09 | <0.001 |
| Right lingual gyrus | 0.84 | 0.10 | <0.001 |
| Right medial orbitofrontal cortex | 0.47 | 0.12 | 0.031 |
| Right middle temporal gyrus | -0.81 | 0.10 | <0.001 |
| Right parahippocampal gyrus | 3.49 | 0.17 | <0.001 |
| Right parsorbitalis | 0.89 | 0.12 | <0.001 |
| Right pars triangularis of inferior frontal gyrus | 1.35 | 0.15 | <0.001 |
| Right posterior cingulate cortex | 0.56 | 0.08 | <0.001 |
| Right precentral gyrus | -0.72 | 0.11 | <0.001 |
| Right rostral middle frontal gyrus | -1.09 | 0.12 | <0.001 |
| Right superior temporal gyrus | -0.96 | 0.13 | <0.001 |
| Right frontal pole | 3.84 | 0.20 | <0.001 |
| Right temporal pole | 0.45 | 0.11 | 0.019 |
| Right transverse temporal gyrus | 2.28 | 0.17 | <0.001 |
| **Deviations from healthy children’s developmental trajectories in paediatric MOGAD patients without brain lesions** | | | |
| **Brain Region** | **Intercept** | **SE** | ****p***  **values** |
| Left caudate nucleus | -1.07 | 0.24 | 0.021 |
| Left pallidum | -1.85 | 0.36 | <0.001 |
| Left nucleus accumbens | -1.30 | 0.30 | 0.032 |
| Right caudate nucleus | -0.98 | 0.23 | 0.034 |
| Right pallidum | -1.70 | 0.36 | 0.009 |
| Left banks of superior temporal sulcus | -0.87 | 0.21 | 0.048 |
| Left lingual gyrus | 0.82 | 0.19 | 0.049 |
| Left pericalcarine cortex | 2.92 | 0.41 | <0.001 |
| Left posterior cingulate cortex | 3.65 | 0.53 | <0.001 |
| Left rostral anterior cingulate cortex | -0.77 | 0.17 | 0.022 |
| Left rostral middle frontal gyrus | -1.68 | 0.27 | <0.001 |
| Left superior temporal gyrus | -1.32 | 0.26 | <0.001 |
| Left supramarginal | -1.11 | 0.19 | <0.001 |
| Left frontal pole | 3.66 | 0.53 | <0.001 |
| Right caudal middle frontal gyrus | 1.37 | 0.24 | <0.001 |
| Right entorhinal cortex | 2.38 | 0.43 | <0.001 |
| Right fusiform gyrus | 2.90 | 0.38 | <0.001 |
| Right inferior temporal gyrus | 1.83 | 0.27 | <0.001 |
| Right parahippocampal gyrus | 2.86 | 0.47 | <0.001 |
| Right rostral middle frontal gyrus | -1.56 | 0.21 | <0.001 |
| Right frontal pole | 3.12 | 0.46 | <0.001 |
| **Deviations from healthy children’s developmental trajectories in paediatric MOGAD with *vs* without brain lesions** | | | |
| **Brain Region** | **β coef** | **SE** | ****p***  **values** |
| Left caudal anterior cingulate cortex | 0.71 | 0.21 | <0.001 |
| Left entorhinal cortex | 0.61 | 0.26 | 0.043 |
| Left inferior parietal lobule | -0.66 | 0.25 | 0.022 |
| Left parahippocampal gyrus | 0.53 | 0.21 | 0.031 |
| Left temporal pole | 0.59 | 0.23 | 0.029 |
| Left transverse temporal gyrus | 0.53 | 0.22 | 0.041 |
| Right caudal anterior cingulate cortex | 0.77 | 0.26 | 0.011 |
| Right cuneus | 0.56 | 0.23 | 0.038 |
| Right lingual gyrus | 0.52 | 0.22 | 0.047 |
| Right parsorbitalis | 0.76 | 0.25 | 0.011 |
| Right pericalcarine cortex | 0.75 | 0.20 | <0.001 |

**p* values reported are corrected for multiple comparison (false discovery rate)

Abbreviations: MOGAD=Myelin Oligodendrocyte Glycoprotein Associated Disease; SE=standard error; coef=coefficient.

**Figure 2.** Summarizes deviations from healthy children’s developmental trajectories at disease onset in paediatric MOGAD patients with complete brain lesion resolution, compared to those of patients with persisting or increasing lesions and those of patients without brain lesions.

Linear mixed-effects models, FDR-corrected p-values <0.001 to 0.048

Intercept

Intercept

**Deviation from age- and sex- expected maturational trajectories at disease onset in paediatric MOGAD patients with complete lesion resolution**

**Table 3.** Summarizes deviations from healthy children’s developmental trajectories at disease onset in paediatric MOGAD patients with complete brain lesion resolution, compared to those of patients with persisting or increasing lesions and those of patients without brain lesions.

| **Deviations from healthy children's developmental trajectories in paediatric MOGAD patients with complete brain lesion resolution** | | | |
| --- | --- | --- | --- |
| **Brain Region** | **Intercept** | **SE** | ****p***  **values** |
| Left entorhinal cortex | 1.45 | 0.18 | <0.001 |
| Left inferior parietal lobule | -1.36 | 0.22 | <0.001 |
| Left inferior temporal gyrus | -0.90 | 0.20 | 0.030 |
| Left lingual gyrus | 0.90 | 0.16 | <0.001 |
| Left middle temporal gyrus | -1.36 | 0.13 | <0.001 |
| Left pars opercularis of inferior frontal gyrus | -0.72 | 0.15 | 0.042 |
| Left pars triangularis of inferior frontal gyrus | -1.08 | 0.24 | 0.041 |
| Left pericalcarine cortex | 3.29 | 0.33 | <0.001 |
| Left postcentral gyrus | -1.52 | 0.21 | <0.001 |
| Left posterior cingulate cortex | 4.29 | 0.42 | <0.001 |
| Left precentral gyrus | -1.42 | 0.19 | <0.001 |
| Left rostral middle frontal gyrus | -1.38 | 0.28 | 0.022 |
| Left superior temporal gyrus | -1.64 | 0.32 | 0.011 |
| Left supramarginal | -1.15 | 0.18 | <0.001 |
| Left frontal pole | 4.43 | 0.43 | <0.001 |
| Right caudal anterior cingulate cortex | 1.38 | 0.19 | <0.001 |
| Right caudal middle frontal gyrus | 1.53 | 0.26 | <0.001 |
| Right entorhinal cortex | 2.71 | 0.29 | <0.001 |
| Right fusiform gyrus | 2.70 | 0.32 | <0.001 |
| Right inferior temporal gyrus | 1.70 | 0.24 | <0.001 |
| Right lingual gyrus | 0.70 | 0.15 | 0.019 |
| Right middle temporal gyrus | -1.05 | 0.18 | <0.001 |
| Right parahippocampal gyrus | 3.32 | 0.41 | <0.001 |
| Right pars triangularis of inferior frontal gyrus | 1.21 | 0.24 | 0.018 |
| Right precentral gyrus | -0.87 | 0.14 | <0.001 |
| Right rostral middle frontal gyrus | -1.04 | 0.21 | 0.021 |
| Right frontal pole | 3.88 | 0.41 | <0.001 |
| Right transverse temporal gyrus | 1.98 | 0.33 | <0.001 |
| **Deviations from healthy children's developmental trajectories in paediatric MOGAD patients with complete brain lesion resolution *vs* with persisting or increasing brain lesions** | | | |
| **Brain Region** | **β coef** | **SE** | ****p***  **values** |
| Left putamen | 1.81 | 0.37 | 0.049 |
| Left pallidum | 1.17 | 0.50 | 0.001 |
| **Deviations from healthy children's developmental trajectories in paediatric MOGAD patients with complete brain lesion resolution *vs* without brain lesions** | | | |
| **Brain Region** | **β coef** | **SE** | ****p***  **values** |
| Left caudate nucleus | -1.04 | 0.35 | 0.046 |
| Right caudate nucleus | -1.07 | 0.34 | 0.031 |
| Right putamen | -1.11 | 0.37 | 0.049 |
| Right pallidum | -1.56 | 0.50 | 0.031 |

**p* values reported are corrected for multiple comparison (false discovery rate)

Abbreviations: MOGAD=Myelin Oligodendrocyte Glycoprotein Associated Disease; SE=standard error; coef=coefficient.

**Figure 3.** Summarizes deviations from healthy children’s developmental trajectories at disease onset in paediatric MOGAD patients with relapsing and monophasic disease course and the comparison between the two groups, grouping patients by the presence of brain lesions at disease onset.

Intercept

Intercept

Intercept

Intercept

Linear mixed-effects models, FDR-corrected p-values <0.001 to 0.05

**Deviation from age- and sex- expected maturational trajectories at disease onset in paediatric MOGAD patients with monophasic and relapsing disease course**

**Table 4.** Summarizes deviations from healthy children’s developmental trajectories at disease onset in paediatric MOGAD patients with relapsing and monophasic disease course and the comparison between the two groups, grouping patients by the presence of brain lesions at disease onset.

| **Deviations from healthy children’s developmental trajectories in paediatric MOGAD patients with monophasic course** | | | | | | | |
| --- | --- | --- | --- | --- | --- | --- | --- |
| **Paediatric MOGAD patients with brain lesions** | | | | **Paediatric MOGAD patients without brain lesions** | | | |
| **Brain Region** | **Intercept** | **SE** | ****p***  **values** | **Brain Region** | **Intercept** | **SE** | ****p***  **values** |
| Left caudate nucleus | -0.88 | 0.21 | <0.001 | Left caudate nucleus | -1.37 | 0.41 | <0.001 |
| Left putamen | -1.32 | 0.23 | <0.001 | Left putamen | -1.49 | 0.35 | <0.001 |
| Left pallidum | -3.15 | 0.35 | <0.001 | Left pallidum | -1.87 | 0.54 | <0.001 |
| Left nucleus accumbens | -1.09 | 0.24 | <0.001 | Left nucleus accumbens | -1.59 | 0.49 | <0.001 |
| Left ventral  diencephalon | 0.71 | 0.23 | 0.011 | Right putamen | -1.64 | 0.40 | <0.001 |
| Right caudate nucleus | -1.02 | 0.22 | <0.001 | Right pallidum | -1.85 | 0.47 | <0.001 |
| Right putamen | -0.96 | 0.21 | <0.001 | Right nucleus accumbens | -1.61 | 0.54 | 0.012 |
| Right pallidum | -1.83 | 0.31 | <0.001 | Left entorhinal cortex | 1.48 | 0.34 | <0.001 |
| Right nucleus accumbens | -0.76 | 0.25 | 0.011 | Left lingual gyrus | 0.80 | 0.23 | <0.001 |
| Right ventral diencephalon | 0.74 | 0.29 | 0.027 | Left middle temporal gyrus | -0.85 | 0.28 | 0.009 |
| Left banks of superior temporal sulcus | -0.73 | 0.17 | <0.001 | Left pars triangularis of inferior frontal gyrus | -1.00 | 0.17 | <0.001 |
| Left caudal middle frontal gyrus | 0.90 | 0.18 | <0.001 | Left pericalcarine cortex | 2.88 | 0.49 | <0.001 |
| Left entorhinal cortex | 1.85 | 0.17 | <0.001 | Left posterior cingulate cortex | 3.46 | 0.62 | <0.001 |
| Left fusiform gyrus | 0.56 | 0.19 | 0.007 | Left rostral middle frontal gyrus | -1.37 | 0.32 | <0.001 |
| Left inferior parietal lobule | -1.54 | 0.17 | <0.001 | Left superior temporal gyrus | -1.31 | 0.28 | <0.001 |
| Left inferior temporal gyrus | -0.58 | 0.18 | <0.001 | Left supramarginal | -1.04 | 0.28 | <0.001 |
| Left lateral occipital gyrus | -0.64 | 0.20 | 0.008 | Left frontal pole | 3.29 | 0.76 | <0.001 |
| Left lateral orbitofrontal cortex | -0.54 | 0.21 | 0.031 | Left temporal pole | 0.70 | 0.22 | 0.007 |
| Left lingual gyrus | 0.93 | 0.15 | <0.001 | Right caudal middle frontal gyrus | 1.32 | 0.24 | <0.001 |
| Left middle temporal gyrus | -1.10 | 0.18 | <0.001 | Right entorhinal cortex | 2.19 | 0.42 | <0.001 |
| Left pars opercularis of inferior frontal gyrus | -0.88 | 0.14 | <0.001 | Right fusiform gyrus | 2.11 | 0.29 | <0.001 |
| Left parsorbitalis | -0.88 | 0.20 | <0.001 | Right inferior temporal gyrus | 1.42 | 0.22 | <0.001 |
| Left pars triangularis of inferior frontal gyrus | -1.03 | 0.17 | <0.001 | Right parahippocampal gyrus | 3.02 | 0.47 | <0.001 |
| Left pericalcarine cortex | 3.74 | 0.26 | <0.001 | Right rostral middle frontal gyrus | -1.45 | 0.30 | <0.001 |
| Left postcentral gyrus | -1.04 | 0.20 | <0.001 | Right frontal pole | 3.13 | 0.55 | <0.001 |
| Left posterior cingulate cortex | 4.88 | 0.34 | <0.001 |  |  |  |  |
| Left precentral gyrus | -0.88 | 0.21 | <0.001 |  |  |  |  |
| Left precuneus | -0.73 | 0.18 | <0.001 |  |  |  |  |
| Left rostral anterior cingulate cortex | -0.49 | 0.16 | 0.009 |  |  |  |  |
| Left rostral middle frontal gyrus | -1.67 | 0.20 | <0.001 |  |  |  |  |
| Left superior temporal gyrus | -0.97 | 0.21 | <0.001 |  |  |  |  |
| Left supramarginal | -1.22 | 0.17 | <0.001 |  |  |  |  |
| Left frontal pole | 4.96 | 0.39 | <0.001 |  |  |  |  |
| Left temporal pole | 1.04 | 0.17 | <0.001 |  |  |  |  |
| Right banks of superior temporal sulcus | 1.35 | 0.21 | <0.001 |  |  |  |  |
| Right caudal anterior cingulate cortex | 1.84 | 0.17 | <0.001 |  |  |  |  |
| Right caudal middle frontal gyrus | 2.21 | 0.19 | <0.001 |  |  |  |  |
| Right entorhinal cortex | 3.47 | 0.22 | <0.001 |  |  |  |  |
| Right fusiform gyrus | 3.59 | 0.26 | <0.001 |  |  |  |  |
| Right inferior temporal gyrus | 2.45 | 0.20 | <0.001 |  |  |  |  |
| Right isthmuscingulate | 1.10 | 0.14 | <0.001 |  |  |  |  |
| Right lingual gyrus | 0.96 | 0.16 | <0.001 |  |  |  |  |
| Right medial orbitofrontal cortex | 0.71 | 0.19 | <0.001 |  |  |  |  |
| Right middle temporal gyrus | -0.61 | 0.17 | <0.001 |  |  |  |  |
| Right parahippocampal gyrus | 3.91 | 0.27 | <0.001 |  |  |  |  |
| Right parsorbitalis | 1.08 | 0.19 | <0.001 |  |  |  |  |
| Right pars triangularis of inferior frontal gyrus | 2.10 | 0.21 | <0.001 |  |  |  |  |
| Right posterior cingulate cortex | 0.66 | 0.13 | <0.001 |  |  |  |  |
| Right precentral gyrus | -0.60 | 0.17 | <0.001 |  |  |  |  |
| Right rostral middle frontal gyrus | -1.25 | 0.20 | <0.001 |  |  |  |  |
| Right superior temporal gyrus | -0.74 | 0.22 | <0.001 |  |  |  |  |
| Right supramarginal | 0.59 | 0.16 | <0.001 |  |  |  |  |
| Right frontal pole | 4.14 | 0.33 | <0.001 |  |  |  |  |
| Right temporal pole | 0.80 | 0.17 | <0.001 |  |  |  |  |
| Right transverse temporal gyrus | 2.90 | 0.26 | <0.001 |  |  |  |  |
| **Deviations from healthy children’s developmental trajectories in paediatric MOGAD patients with relapsing course** | | | | | | | |
| **Paediatric MOGAD patients with brain lesions** | | | | **Paediatric MOGAD patients without brain lesions** | | | |
| **Brain Region** | **Intercept** | **SE** | ****p***  **values** | **Brain Region** | **Intercept** | **SE** | ****p***  **values** |
| Left caudate nucleus | -0.87 | 0.20 | <0.001 | Left pallidum | -1.83 | 0.49 | <0.001 |
| Left putamen | -1.32 | 0.27 | <0.001 | Right caudate nucleus | -0.93 | 0.28 | 0.011 |
| Left pallidum | -3.15 | 0.39 | <0.001 | Left banks of superior temporal sulcus | -0.84 | 0.27 | 0.013 |
| Left nucleus accumbens | -1.09 | 0.26 | <0.001 | Left caudal middle frontal gyrus | 0.90 | 0.16 | <0.001 |
| Left ventral diencephalon | 0.71 | 0.28 | 0.021 | Left inferior temporal gyrus | -0.97 | 0.31 | 0.008 |
| Right caudate nucleus | -1.02 | 0.24 | <0.001 | Left pericalcarine cortex | 2.97 | 0.66 | <0.001 |
| Right putamen | -0.97 | 0.25 | <0.001 | Left postcentral gyrus | -1.05 | 0.35 | 0.009 |
| Right pallidum | -1.83 | 0.36 | <0.001 | Left posterior cingulate cortex | 3.85 | 0.87 | <0.001 |
| Right nucleus accumbens | -0.76 | 0.28 | 0.011 | Left rostral anterior cingulate cortex | -1.06 | 0.24 | <0.001 |
| Right ventral diencephalon | 0.74 | 0.34 | 0.048 | Left rostral middle frontal gyrus | -1.99 | 0.40 | <0.001 |
| Left banks of superior temporal sulcus | -0.74 | 0.12 | <0.001 | Left superior frontal gyrus | -1.65 | 0.37 | <0.001 |
| Left caudal anterior cingulate cortex | 0.39 | 0.15 | 0.021 | Left superior temporal gyrus | -1.37 | 0.43 | 0.027 |
| Left caudal middle frontal gyrus | 0.90 | 0.17 | <0.001 | Left supramarginal | -1.13 | 0.24 | <0.001 |
| Left entorhinal cortex | 1.84 | 0.14 | <0.001 | Left frontal pole | 4.03 | 0.74 | <0.001 |
| Left fusiform gyrus | 0.57 | 0.19 | 0.012 | Right caudal middle frontal gyrus | 1.46 | 0.43 | <0.001 |
| Left inferior parietal lobule | -1.54 | 0.20 | <0.001 | Right cuneus | -1.00 | 0.33 | 0.032 |
| Left inferior temporal gyrus | -0.58 | 0.20 | 0.010 | Right entorhinal cortex | 2.55 | 0.76 | <0.001 |
| Left lateral occipital gyrus | -0.65 | 0.19 | <0.001 | Right fusiform gyrus | 3.66 | 0.67 | <0.001 |
| Left lateral orbitofrontal cortex | -0.55 | 0.17 | 0.011 | Right inferior temporal gyrus | 2.26 | 0.50 | <0.001 |
| Left lingual gyrus | 0.94 | 0.16 | <0.001 | Right parahippocampal gyrus | 2.75 | 0.80 | <0.001 |
| Left middle temporal gyrus | -1.10 | 0.20 | <0.001 | Right pars triangularis of inferior frontal gyrus | 1.24 | 0.35 | <0.001 |
| Left pars opercularis of inferior frontal gyrus | -0.88 | 0.14 | <0.001 | Right rostral middle frontal gyrus | -1.68 | 0.30 | <0.001 |
| Left parsorbitalis | -0.88 | 0.21 | <0.001 | Right superior frontal gyrus | -0.71 | 0.23 | 0.011 |
| Left pars triangularis of inferior frontal gyrus | -1.03 | 0.16 | <0.001 | Right supramarginal | 0.66 | 0.18 | <0.001 |
| Left pericalcarine cortex | 3.74 | 0.24 | <0.001 | Right frontal pole | 3.14 | 0.75 | <0.001 |
| Left postcentral gyrus | -1.03 | 0.23 | <0.001 |  |  |  |  |
| Left posterior cingulate cortex | 4.88 | 0.29 | <0.001 |  |  |  |  |
| Left precentral gyrus | -0.88 | 0.22 | <0.001 |  |  |  |  |
| Left precuneus | -0.73 | 0.17 | <0.001 |  |  |  |  |
| Left rostral anterior cingulate cortex | -0.49 | 0.17 | 0.009 |  |  |  |  |
| Left rostral middle frontal gyrus | -1.67 | 0.19 | <0.001 |  |  |  |  |
| Left superior frontal gyrus | -0.47 | 0.21 | 0.041 |  |  |  |  |
| Left superiorparietal | -0.27 | 0.12 | 0.039 |  |  |  |  |
| Left superior temporal gyrus | -0.97 | 0.16 | <0.001 |  |  |  |  |
| Left supramarginal | -1.22 | 0.16 | <0.001 |  |  |  |  |
| Left frontal pole | 4.97 | 0.37 | <0.001 |  |  |  |  |
| Left temporal pole | 1.03 | 0.16 | <0.001 |  |  |  |  |
| Right banks of superior temporal sulcus | 1.36 | 0.20 | <0.001 |  |  |  |  |
| Right caudal anterior cingulate cortex | 1.83 | 0.15 | <0.001 |  |  |  |  |
| Right caudal middle frontal gyrus | 2.22 | 0.19 | <0.001 |  |  |  |  |
| Right entorhinal cortex | 3.48 | 0.20 | <0.001 |  |  |  |  |
| Right fusiform gyrus | 3.59 | 0.26 | <0.001 |  |  |  |  |
| Right inferior temporal gyrus | 2.45 | 0.23 | <0.001 |  |  |  |  |
| Right isthmuscingulate | 1.10 | 0.14 | <0.001 |  |  |  |  |
| Right lateral occipital gyrus | -0.37 | 0.15 | 0.031 |  |  |  |  |
| Right lateral orbitofrontal cortex | 0.42 | 0.19 | 0.049 |  |  |  |  |
| Right lingual gyrus | 0.97 | 0.19 | <0.001 |  |  |  |  |
| Right medial orbitofrontal cortex | 0.71 | 0.20 | <0.001 |  |  |  |  |
| Right middle temporal gyrus | -0.61 | 0.17 | <0.001 |  |  |  |  |
| Right parahippocampal gyrus | 3.91 | 0.19 | <0.001 |  |  |  |  |
| Right paracentral lobule | 0.35 | 0.16 | 0.050 |  |  |  |  |
| Right parsorbitalis | 1.07 | 0.18 | <0.001 |  |  |  |  |
| Right pars triangularis of inferior frontal gyrus | 2.10 | 0.21 | <0.001 |  |  |  |  |
| Right posterior cingulate cortex | 0.65 | 0.12 | <0.001 |  |  |  |  |
| Right precentral gyrus | -0.60 | 0.17 | <0.001 |  |  |  |  |
| Right rostral middle frontal gyrus | -1.25 | 0.20 | <0.001 |  |  |  |  |
| Right superior temporal gyrus | -0.75 | 0.18 | <0.001 |  |  |  |  |
| Right supramarginal | 0.59 | 0.14 | <0.001 |  |  |  |  |
| Right frontal pole | 4.14 | 0.30 | <0.001 |  |  |  |  |
| Right temporal pole | 0.81 | 0.15 | <0.001 |  |  |  |  |
| Right transverse temporal gyrus | 2.90 | 0.27 | <0.001 |  |  |  |  |
| **Deviations from healthy children’s developmental trajectories in paediatric MOGAD patients with relapsing *vs* monophasic course** | | | | | | | |
| **Paediatric MOGAD patients with brain lesions** | | | | **Paediatric MOGAD patients without brain lesions** | | | |
| **Brain Region** | **β coef** | **SE** | ****p***  **values** | **Brain Region** | **β coef** | **SE** | ****p***  **values** |
| Left pallidum | -2.31 | 0.45 | <0.001 |  |  |  |  |
| Right caudate nucleus | -0.75 | 0.28 | 0.047 |  |  |  |  |
| Right pallidum | -1.23 | 0.40 | 0.024 |  |  |  |  |
| Left parsorbitalis | -0.75 | 0.25 | 0.001 |  |  |  |  |
| Right caudal middle frontal gyrus | 0.71 | 0.24 | 0.017 |  |  |  |  |
| Right entorhinal cortex | 0.90 | 0.28 | 0.002 |  |  |  |  |
| Right fusiform gyrus | 1.18 | 0.34 | <0.001 |  |  |  |  |
| Right inferior temporal gyrus | 1.14 | 0.25 | <0.001 |  |  |  |  |
| Right isthmuscingulate | 0.43 | 0.18 | 0.047 |  |  |  |  |
| Right paracentral lobule | 0.54 | 0.19 | 0.025 |  |  |  |  |
| Right pars triangularis of inferior frontal gyrus | 1.22 | 0.27 | <0.001 |  |  |  |  |
| Right postcentral gyrus | 0.63 | 0.22 | 0.039 |  |  |  |  |
| Right temporal pole | 0.55 | 0.21 | 0.034 |  |  |  |  |
| Right transverse temporal gyrus | 1.02 | 0.33 | 0.003 |  |  |  |  |
| Right insula | 0.64 | 0.26 | 0.042 |  |  |  |  |

**p* values reported are corrected for multiple comparison (false discovery rate)

Abbreviations: MOGAD=Myelin Oligodendrocyte Glycoprotein Associated Disease; SE=standard error; coef=coefficient.

**Figure 4.** Summarizes changes over time of deviations from healthy children’s developmental trajectories in paediatric MOGAD patients with and without brain lesions and the comparison between the two groups.

Linear mixed-effects models, FDR-corrected p-values <0.001 to 0.043

**Table 5.** Summarizes changes over time of deviations from healthy children’s developmental trajectories in paediatric MOGAD patients with and without brain lesions and the comparison between the two groups.

| **Deviations from healthy children’s developmental trajectories in paediatric MOGAD patients with brain lesions** | | | |
| --- | --- | --- | --- |
| **Brain Region** | **β coef** | **SE** | ****p***  **values** |
| Left thalamus | -0.47 | 0.06 | <0.001 |
| Left nucleus accumbens | -0.15 | 0.04 | 0.021 |
| Left ventral diencephalon | -0.27 | 0.04 | <0.001 |
| Right thalamus | -0.42 | 0.05 | <0.001 |
| Right putamen | -0.16 | 0.03 | <0.001 |
| Right ventral diencephalon | -0.33 | 0.05 | <0.001 |
| Left banks of superior temporal sulcus | -0.08 | 0.02 | <0.001 |
| Left caudal anterior cingulate cortex | -0.14 | 0.01 | <0.001 |
| Left caudal middle frontal gyrus | -0.07 | 0.02 | <0.001 |
| Left cuneus | -0.08 | 0.02 | <0.001 |
| Left entorhinal cortex | -0.09 | 0.01 | <0.001 |
| Left inferior temporal gyrus | -0.12 | 0.02 | <0.001 |
| Left lateral occipital gyrus | -0.07 | 0.02 | 0.042 |
| Left lateral orbitofrontal cortex | -0.11 | 0.02 | <0.001 |
| Left medial orbitofrontal cortex | -0.10 | 0.02 | <0.001 |
| Left parahippocampal gyrus | -0.17 | 0.01 | <0.001 |
| Left paracentral lobule | -0.08 | 0.02 | 0.031 |
| Left pars opercularis of inferior frontal gyrus | -0.10 | 0.02 | <0.001 |
| Left pars triangularis of inferior frontal gyrus | -0.07 | 0.01 | <0.001 |
| Left pericalcarine cortex | -0.08 | 0.01 | <0.001 |
| Left posterior cingulate cortex | -0.09 | 0.02 | <0.001 |
| Left precentral gyrus | -0.13 | 0.02 | <0.001 |
| Left rostral anterior cingulate cortex | -0.10 | 0.02 | <0.001 |
| Left superior frontal gyrus | -0.16 | 0.02 | <0.001 |
| Left temporal pole | -0.13 | 0.01 | <0.001 |
| Left transverse temporal gyrus | -0.14 | 0.01 | <0.001 |
| Left insula | -0.19 | 0.03 | <0.001 |
| Right banks of superior temporal sulcus | -0.10 | 0.02 | <0.001 |
| Right caudal anterior cingulate cortex | -0.09 | 0.01 | <0.001 |
| Right caudal middle frontal gyrus | -0.08 | 0.02 | <0.001 |
| Right entorhinal cortex | -0.15 | 0.02 | <0.001 |
| Right fusiform gyrus | -0.14 | 0.02 | <0.001 |
| Right inferior temporal gyrus | -0.13 | 0.02 | <0.001 |
| Right isthmuscingulate | -0.06 | 0.01 | <0.001 |
| Right lateral orbitofrontal cortex | -0.18 | 0.02 | <0.001 |
| Right lingual gyrus | -0.10 | 0.01 | <0.001 |
| Right medial orbitofrontal cortex | -0.11 | 0.02 | <0.001 |
| Right middle temporal gyrus | -0.10 | 0.02 | <0.001 |
| Right parahippocampal gyrus | -0.17 | 0.02 | <0.001 |
| Right paracentral lobule | -0.10 | 0.02 | <0.001 |
| Right pars opercularis of inferior frontal gyrus | -0.09 | 0.02 | <0.001 |
| Right parsorbitalis | -0.18 | 0.02 | <0.001 |
| Right pars triangularis of inferior frontal gyrus | -0.15 | 0.02 | <0.001 |
| Right pericalcarine cortex | -0.14 | 0.02 | <0.001 |
| Right rostral anterior cingulate cortex | -0.08 | 0.01 | <0.001 |
| Right superior frontal gyrus | -0.12 | 0.02 | <0.001 |
| Right superior temporal gyrus | -0.11 | 0.02 | <0.001 |
| Right frontal pole | -0.08 | 0.02 | <0.001 |
| Right temporal pole | -0.16 | 0.02 | <0.001 |
| Right transverse temporal gyrus | -0.18 | 0.02 | <0.001 |
| Right insula | -0.19 | 0.02 | <0.001 |
| **Deviations from healthy children’s developmental trajectories in paediatric MOGAD with *vs* without brain lesions** | | | |
| **Brain Region** | **β coef** | **SE** | ****p***  **values** |
| Right thalamus | -0.44 | 0.14 | 0.011 |
| Right putamen | -0.19 | 0.08 | 0.041 |
| Left banks of superior temporal sulcus | -0.14 | 0.05 | 0.019 |
| Left cuneus | -0.14 | 0.04 | 0.009 |
| Left entorhinal cortex | -0.13 | 0.04 | <0.001 |
| Left parahippocampal gyrus | -0.16 | 0.05 | <0.001 |
| Left pericalcarine cortex | -0.12 | 0.04 | 0.008 |
| Left posterior cingulate cortex | -0.15 | 0.05 | 0.009 |
| Left supramarginal | -0.16 | 0.06 | 0.021 |
| Left temporal pole | -0.12 | 0.04 | <0.001 |
| Left transverse temporal gyrus | -0.10 | 0.04 | 0.043 |
| Right banks of superior temporal sulcus | -0.15 | 0.05 | 0.011 |
| Right caudal anterior cingulate cortex | -0.13 | 0.04 | <0.001 |
| Right cuneus | -0.20 | 0.06 | <0.001 |
| Right entorhinal cortex | -0.12 | 0.04 | 0.021 |
| Right inferior temporal gyrus | -0.14 | 0.06 | 0.034 |
| Right parahippocampal gyrus | -0.16 | 0.05 | 0.011 |
| Right parsorbitalis | -0.15 | 0.05 | 0.009 |
| Right pars triangularis of inferior frontal gyrus | -0.12 | 0.05 | 0.009 |
| Right pericalcarine cortex | -0.17 | 0.05 | <0.001 |
| Right posterior cingulate cortex | -0.14 | 0.04 | <0.001 |
| Right rostral middle frontal gyrus | -0.13 | 0.05 | 0.012 |
| Right supramarginal | -0.17 | 0.05 | <0.001 |
| Right frontal pole | -0.17 | 0.06 | 0.010 |
| Right transverse temporal gyrus | -0.20 | 0.05 | <0.001 |

**p* values reported are corrected for multiple comparison (false discovery rate)

Abbreviations: MOGAD=Myelin Oligodendrocyte Glycoprotein Associated Disease; SE=standard error; coef=coefficient.

**Figure 5.** Summarizes changes over time of deviations from healthy children’s developmental trajectories in paediatric MOGAD patients with complete lesion resolution, compared to those of patients with persisting or increasing brain lesions and patients without brain lesions.

Linear mixed-effects models, FDR-corrected p-values <0.001 to 0.049

**Table 6.** Summarizes changes over time of deviations from healthy children’s developmental trajectories in paediatric MOGAD patients with complete lesion resolution, compared to those of patients with persisting or increasing brain lesions.

| **Deviations from healthy children's developmental trajectories in paediatric MOGAD patients with resolving lesions** | | | |
| --- | --- | --- | --- |
| **Brain Region** | **β coef** | **SE** | ****p***  **values** |
| Left caudal anterior cingulate cortex | -0.26 | 0.05 | <0.001 |
| Left parahippocampal gyrus | -0.24 | 0.05 | 0.031 |
| Right lateral orbitofrontal cortex | -0.31 | 0.07 | 0.048 |
| **Deviations from healthy children's developmental trajectories in paediatric MOGAD patients with resolving lesions *vs* persisting increasing lesions** | | | |
| **Brain Region** | **β coef** | **SE** | ****p***  **values** |
| Left caudal anterior cingulate cortex | -0.13 | 0.07 | 0.047 |
| Left supramarginal gyrus | 0.18 | 0.08 | 0.048 |
| Right inferior parietal gyrus | 0.17 | 0.08 | 0.049 |
| Right insula | -0.25 | 0.12 | 0.048 |

**p* values reported are corrected for multiple comparison (false discovery rate)

Abbreviations: MOGAD=Myelin Oligodendrocyte Glycoprotein Associated Disease; SE=standard error; coef=coefficient.

**Figure 6.** Summarizes changes over time of deviations from healthy children’s developmental trajectories at in paediatric MOGAD patients with relapsing and monophasic disease course and the comparison between the two groups, grouping patients by the presence of brain lesions at disease onset.

Linear mixed-effects models, FDR-corrected p-values <0.001 to 0.049

**Table 7.** Summarizes changes over time of deviations from healthy children’s developmental trajectories in paediatric MOGAD patients with monophasic and relapsing course at disease onset grouped according to the presence of brain MRI lesions at disease onset.

| **Deviations from healthy children’s developmental trajectories in paediatric MOGAD patients with monophasic course** | | | | | | | |
| --- | --- | --- | --- | --- | --- | --- | --- |
| **Paediatric MOGAD patients with brain lesions** | | | | **Paediatric MOGAD patients without brain lesions** | | | |
| **Brain Region** | **β coef** | **SE** | ****p***  **values** | **Brain Region** | **β coef** | **SE** | ****p***  **values** |
| Left ventral diencephalon | -0.22 | 0.08 | 0.021 |  |  |  |  |
| Right-Thalamus-Proper | -0.35 | 0.12 | 0.009 |  |  |  |  |
| Right-Putamen | -0.21 | 0.09 | 0.046 |  |  |  |  |
| Rightventral diencephalon | -0.28 | 0.11 | 0.031 |  |  |  |  |
| Left lateral orbitofrontal cortex | -0.15 | 0.05 | 0.019 |  |  |  |  |
| Left lingual gyrus | 0.09 | 0.03 | 0.009 |  |  |  |  |
| Left parahippocampal gyrus | -0.15 | 0.03 | <0.001 |  |  |  |  |
| Left transverse temporal gyrus | -0.10 | 0.04 | 0.046 |  |  |  |  |
| Left insula | -0.23 | 0.07 | <0.001 |  |  |  |  |
| Right entorhinal cortex | -0.10 | 0.04 | 0.021 |  |  |  |  |
| Right lateral orbitofrontal cortex | -0.22 | 0.06 | <0.001 |  |  |  |  |
| Right parahippocampal gyrus | -0.13 | 0.05 | 0.041 |  |  |  |  |
| Right pericalcarine cortex | -0.14 | 0.06 | 0.048 |  |  |  |  |
| Right temporal pole | -0.20 | 0.04 | <0.001 |  |  |  |  |
| Right insula | -0.20 | 0.08 | 0.049 |  |  |  |  |
| Left ventral diencephalon | -0.22 | 0.08 | 0.021 |  |  |  |  |
| Right-Thalamus-Proper | -0.35 | 0.12 | 0.010 |  |  |  |  |
| Right-Putamen | -0.21 | 0.09 | 0.047 |  |  |  |  |
| Rightventral diencephalon | -0.28 | 0.11 | 0.031 |  |  |  |  |
| Left lateral orbitofrontal cortex | -0.15 | 0.05 | 0.021 |  |  |  |  |
| Left lingual gyrus | 0.09 | 0.03 | 0.008 |  |  |  |  |
| Left parahippocampal gyrus | -0.15 | 0.03 | <0.001 |  |  |  |  |
| Left transverse temporal gyrus | -0.10 | 0.04 | 0.049 |  |  |  |  |
| Left insula | -0.23 | 0.07 | <0.001 |  |  |  |  |
| Right entorhinal cortex | -0.10 | 0.04 | 0.021 |  |  |  |  |
| Right lateral orbitofrontal cortex | -0.22 | 0.06 | <0.001 |  |  |  |  |
| Right parahippocampal gyrus | -0.13 | 0.05 | 0.037 |  |  |  |  |
| Right pericalcarine cortex | -0.14 | 0.06 | 0.048 |  |  |  |  |
| Right temporal pole | -0.20 | 0.04 | <0.001 |  |  |  |  |
| Right insula | -0.20 | 0.08 | 0.044 |  |  |  |  |
| **Deviations from healthy children’s developmental trajectories in paediatric MOGAD patients with relapsing course** | | | | | | | |
| **Paediatric MOGAD patients with brain lesions** | | | | **Paediatric MOGAD patients without brain lesions** | | | |
| **Brain Region** | **β coef** | **SE** | ****p***  **values** | **Brain Region** | **β coef** | **SE** | ****p***  **values** |
| Left thalamus | -0.51 | 0.08 | <0.001 | Left thalamus | -0.37 | 0.12 | 0.021 |
| Left putamen | -0.12 | 0.05 | 0.019 | Right thalamus | -0.25 | 0.09 | 0.039 |
| Left hippocampus | -0.09 | 0.04 | 0.031 | Left caudal anterior cingulate cortex | -0.10 | 0.04 | 0.041 |
| Left amygdala | -0.13 | 0.04 | 0.007 | Left entorhinal cortex | -0.05 | 0.02 | 0.042 |
| Left nucleus accumbens | -0.14 | 0.05 | 0.004 | Left fusiform gyrus | -0.16 | 0.04 | 0.008 |
| Left ventral diencephalon | -0.30 | 0.05 | <0.001 | Left lateral orbitofrontal cortex | -0.13 | 0.04 | 0.019 |
| Right thalamus | -0.41 | 0.07 | <0.001 | Left pars triangularis of inferior frontal gyrus | -0.07 | 0.02 | 0.009 |
| Right putamen | -0.14 | 0.03 | <0.001 | Left rostral anterior cingulate cortex | -0.12 | 0.04 | 0.008 |
| Right hippocampus | -0.12 | 0.04 | <0.001 | Left temporal pole | -0.08 | 0.02 | <0.001 |
| Right-amygdala | -0.13 | 0.04 | 0.005 | Left insula | -0.17 | 0.03 | <0.001 |
| Right nucleus accumbens | -0.12 | 0.04 | 0.008 | Right entorhinal cortex | -0.10 | 0.02 | <0.001 |
| Right ventral diencephalon | -0.36 | 0.06 | <0.001 | Right lateral orbitofrontal cortex | -0.16 | 0.04 | 0.009 |
| Left banks of superior temporal sulcus | -0.09 | 0.02 | <0.001 | Right parahippocampal gyrus | -0.07 | 0.02 | 0.008 |
| Left caudal anterior cingulate cortex | -0.15 | 0.01 | <0.001 | Right pars opercularis of inferior frontal gyrus | -0.08 | 0.03 | 0.022 |
| Left caudal middle frontal gyrus | -0.09 | 0.02 | <0.001 | Right superiorparietal | -0.10 | 0.03 | 0.031 |
| Left cuneus | -0.09 | 0.02 | <0.001 | Right temporal pole | -0.18 | 0.04 | <0.001 |
| Left entorhinal cortex | -0.09 | 0.02 | <0.001 | Right insula | -0.18 | 0.05 | 0.011 |
| Left fusiform gyrus | -0.12 | 0.03 | <0.001 |  |  |  |  |
| Left inferior temporal gyrus | -0.14 | 0.03 | <0.001 |  |  |  |  |
| Left isthmuscingulate | -0.10 | 0.03 | <0.001 |  |  |  |  |
| Left lateral occipital gyrus | -0.07 | 0.02 | <0.001 |  |  |  |  |
| Left lateral orbitofrontal cortex | -0.10 | 0.03 | <0.001 |  |  |  |  |
| Left lingual gyrus | 0.03 | 0.01 | 0.034 |  |  |  |  |
| Left medial orbitofrontal cortex | -0.11 | 0.03 | <0.001 |  |  |  |  |
| Left middle temporal gyrus | -0.09 | 0.03 | <0.001 |  |  |  |  |
| Left parahippocampal gyrus | -0.17 | 0.02 | <0.001 |  |  |  |  |
| Left paracentral lobule | -0.08 | 0.02 | <0.001 |  |  |  |  |
| Left pars opercularis of inferior frontal gyrus | -0.10 | 0.02 | <0.001 |  |  |  |  |
| Left pars triangularis of inferior frontal gyrus | -0.07 | 0.01 | <0.001 |  |  |  |  |
| Left pericalcarine cortex | -0.09 | 0.01 | <0.001 |  |  |  |  |
| Left posterior cingulate cortex | -0.11 | 0.02 | <0.001 |  |  |  |  |
| Left precentral gyrus | -0.15 | 0.03 | <0.001 |  |  |  |  |
| Left rostral anterior cingulate cortex | -0.11 | 0.02 | <0.001 |  |  |  |  |
| Left superior frontal gyrus | -0.17 | 0.02 | <0.001 |  |  |  |  |
| Left superior temporal gyrus | -0.06 | 0.03 | 0.046 |  |  |  |  |
| Left frontal pole | -0.07 | 0.03 | 0.033 |  |  |  |  |
| Left temporal pole | -0.14 | 0.02 | <0.001 |  |  |  |  |
| Left transverse temporal gyrus | -0.15 | 0.01 | <0.001 |  |  |  |  |
| Left insula | -0.20 | 0.03 | <0.001 |  |  |  |  |
| Right banks of superior temporal sulcus | -0.12 | 0.02 | <0.001 |  |  |  |  |
| Right caudal anterior cingulate cortex | -0.10 | 0.02 | <0.001 |  |  |  |  |
| Right caudal middle frontal gyrus | -0.10 | 0.02 | <0.001 |  |  |  |  |
| Right cuneus | -0.08 | 0.02 | <0.001 |  |  |  |  |
| Right entorhinal cortex | -0.16 | 0.02 | <0.001 |  |  |  |  |
| Right fusiform gyrus | -0.18 | 0.02 | <0.001 |  |  |  |  |
| Right inferiorparietal | -0.07 | 0.02 | <0.001 |  |  |  |  |
| Right inferior temporal gyrus | -0.17 | 0.02 | <0.001 |  |  |  |  |
| Right isthmuscingulate | -0.08 | 0.02 | <0.001 |  |  |  |  |
| Right lateral occipital gyrus | -0.06 | 0.02 | <0.001 |  |  |  |  |
| Right lateral orbitofrontal cortex | -0.19 | 0.03 | <0.001 |  |  |  |  |
| Right lingual gyrus | -0.11 | 0.02 | <0.001 |  |  |  |  |
| Right medial orbitofrontal cortex | -0.12 | 0.03 | <0.001 |  |  |  |  |
| Right middle temporal gyrus | -0.12 | 0.02 | <0.001 |  |  |  |  |
| Right parahippocampal gyrus | -0.17 | 0.02 | <0.001 |  |  |  |  |
| Right paracentral lobule | -0.12 | 0.02 | <0.001 |  |  |  |  |
| Right pars opercularis of inferior frontal gyrus | -0.09 | 0.02 | <0.001 |  |  |  |  |
| Right parsorbitalis | -0.19 | 0.02 | <0.001 |  |  |  |  |
| Right pars triangularis of inferior frontal gyrus | -0.17 | 0.02 | <0.001 |  |  |  |  |
| Right pericalcarine cortex | -0.14 | 0.02 | <0.001 |  |  |  |  |
| Right postcentral gyrus | -0.10 | 0.02 | <0.001 |  |  |  |  |
| Right precentral gyrus | -0.08 | 0.02 | <0.001 |  |  |  |  |
| Right precuneus | -0.11 | 0.03 | <0.001 |  |  |  |  |
| Right rostral anterior cingulate cortex | -0.08 | 0.02 | <0.001 |  |  |  |  |
| Right rostral middle frontal gyrus | -0.05 | 0.02 | 0.009 |  |  |  |  |
| Right superior frontal gyrus | -0.14 | 0.02 | <0.001 |  |  |  |  |
| Right superiorparietal | -0.06 | 0.02 | <0.001 |  |  |  |  |
| Right superior temporal gyrus | -0.13 | 0.02 | <0.001 |  |  |  |  |
| Right supramarginal | -0.08 | 0.02 | <0.001 |  |  |  |  |
| Right frontal pole | -0.08 | 0.02 | <0.001 |  |  |  |  |
| Right temporal pole | -0.17 | 0.02 | <0.001 |  |  |  |  |
| Right transverse temporal gyrus | -0.20 | 0.02 | <0.001 |  |  |  |  |
| Right insula | -0.20 | 0.02 | <0.001 |  |  |  |  |
| **Deviations from healthy children’s developmental trajectories in paediatric MOGAD patients with relapsing *vs* monophasic course** | | | | | | | |
| **Paediatric MOGAD patients with brain lesions** | | | | **Paediatric MOGAD patients without brain lesions** | | | |
| **Brain Region** | **β coef** | **SE** | ****p***  **values** | **Brain Region** | **β coef** | **SE** | ****p***  **values** |
| Left caudal middle frontal gyrus | -0.13 | 0.05 | 0.022 | Left entorhinal cortex | -0.29 | 0.09 | 0.031 |
| Left fusiform gyrus | -0.18 | 0.07 | 0.035 | Left temporal pole | -0.23 | 0.07 | 0.029 |
| Left supramarginal | -0.14 | 0.06 | 0.044 | Right caudal anterior cingulate cortex | -0.22 | 0.07 | 0.042 |
| Right caudal anterior cingulate cortex | -0.11 | 0.04 | 0.029 | Right pars opercularis of inferior frontal gyrus | -0.24 | 0.07 | 0.028 |
| Right fusiform gyrus | -0.19 | 0.06 | 0.006 | Right posterior cingulate cortex | -0.31 | 0.10 | 0.038 |
| Right inferior temporal gyrus | -0.21 | 0.06 | 0.003 | Right superior temporal gyrus | -0.28 | 0.09 | 0.034 |
| Right paracentral lobule | -0.15 | 0.06 | 0.027 | Right supramarginal | -0.32 | 0.10 | 0.031 |
| Right postcentral gyrus | -0.19 | 0.06 | 0.011 | Right insula | -0.42 | 0.13 | 0.028 |
| Right precuneus | -0.24 | 0.07 | 0.002 |  |  |  |  |
| Right superiorparietal | -0.17 | 0.05 | 0.008 |  |  |  |  |

**p* values reported are corrected for multiple comparison (false discovery rate)

Abbreviations: MOGAD=Myelin Oligodendrocyte Glycoprotein Associated Disease; SE=standard error; coef=coefficient.
